# A stress-blinded Atf1 can fully assemble heterochromatin in a RNAi-independent minimal mat locus but impairs directionality of *mat2/3* switching

**DOI:** 10.1016/j.isci.2022.104820

**Published:** 2022-08-02

**Authors:** Rodrigo Fraile, Laura Sánchez-Mir, Guillem Murciano-Julià, José Ayté, Elena Hidalgo

**Affiliations:** 1 Oxidative Stress and Cell Cycle Group, Universitat Pompeu Fabra, C/ Dr. Aiguader 88, 08003 Barcelona, Spain

**Keywords:** Molecular biology, Molecular mechanism of gene regulation, Epigenetics, Functional aspects of cell biology

## Abstract

The MAP kinase Sty1 phosphorylates and activates the transcription factor Atf1 in response to several stress conditions, which then shifts from a transcriptional repressor to an activator. Atf1 also participates in heterochromatin assembly at the *mat* locus, in combination with the RNA interference (RNAi) machinery. Here, we study the role of signal-dependent phosphorylation of Atf1 in heterochromatin establishment at *mat*, using different Atf1 phospho mutants. Although a hypo-phosphorylation Atf1 mutant, Atf1.10M, mediates heterochromatin assembly, the phosphomimic Atf1.10D is unable to maintain silencing. In a minimal *mat* locus, lacking the RNAi-recruiting *cis* elements and displaying intermediate silencing, Atf1.10M restores full heterochromatin and silencing. However, evolution experiments with this stress-blinded Atf1.10M show that it is unable to facilitate switching between the donor site *mat3* and *mat1*. We propose that the unphosphorylated, inactive Atf1 contributes to proper heterochromatin assembly by recruiting repressive complexes, but its stress-dependent phosphorylation is required for recombination/switching to occur.

## Highlights

• The phosphorylation domain of Atf1 TF is required for heterochromatin assembly at *mat*
• Hypo-phosphorylated Atf1.10M mediates silencing by recruiting repressive complexes
• Stress-dependent phosphorylation of Atf1 is required for recombination and switching
• Atf1.10M is a heterochromatin assembly factor but impairs *mat2/3* switching

## Graphical abstract

**Figure:**
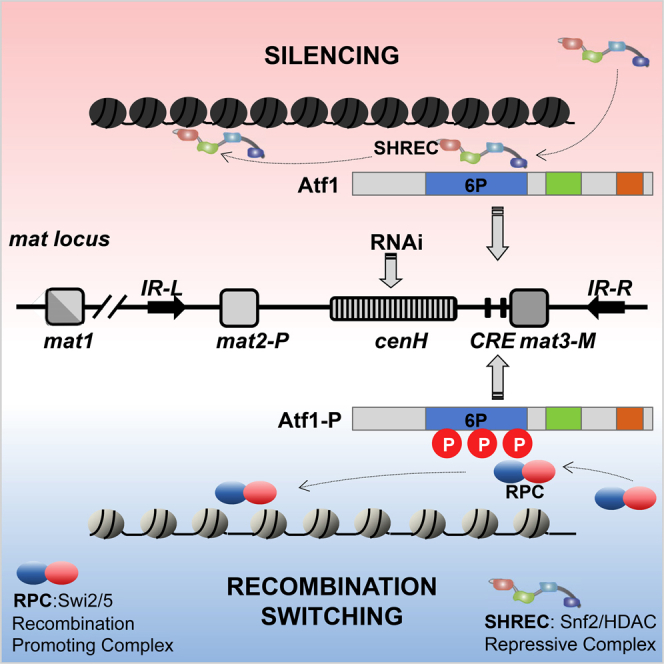

## Introduction

Heterochromatin in eukaryotic chromosomes plays central roles in transcription silencing, maintenance of genome integrity, and chromosome separation, among others. It is generated through the recruitment of DNA- and histone-modifying complexes to initiate heterochromatin sites, which then are spread and epigenetically inherited (Grewal and Jia, 2007; Piunti and Shilatifard, 2016; Richards and Elgin, 2002). Defining how heterochromatin domains are assembled and propagated is essential for understanding normal development and cell physiology. In *Schizosaccharomyces pombe*, heterochromatin is present in three main regions: at centromeres, essential for the accurate chromosome segregation in mitosis; at telomeres, to protect chromosomes from degradation and from aberrant recombination events; and at the mating-type region, to facilitate the correct sequence exchange (switching) between the *mat2* and *mat3* genes with the *mat1* locus, located outside the heterochromatic area [for reviews, see (Allshire and Ekwall, 2015; Grewal and Jia, 2007; Martienssen and Moazed, 2015; Mizuguchi et al., 2015; Thon et al., 2019)]. The insertion of reporter genes in these three heterochromatin areas results in transcriptional repression or silencing of the otherwise euchromatic genes (Allshire et al., 1994; Lorentz et al., 1992; Nimmo et al., 1994).

The mating-type (*mat*) locus is a 20-kb-long silent domain, surrounded by the boundary elements *IR-L* and *IR-R* (Noma et al., 2001). The *mat* locus includes the *mat2* and *mat3* donor loci and the interval between them, the *K-*region [for a review, see (Klar et al., 2014)]. Heterochromatin assembly at this domain mediates silencing in the area and recombinational suppression, and promotes directionality of switching between *mat2* or *mat3* with the acceptor *mat1* locus, a process known as mating-type switching.

Directionality of switching in *S*. *pombe*, by which an *h*^*90*^ culture has half the population carrying the *mat1-P* (*mat2*) information and half the *mat1-M* (*mat3*) gene, is mainly ruled by two features: heterochromatin formation, which establishes structural constraints to the mating-type switching approaching *mat2* to *mat1*, and the Swi2/5 recombination-promoting complex, which accumulates at the *mat3* boundary between these loci and *IR-R*, and ensures that this unfavorably located locus is the donor in *mat1-P*-containing cells (Jia et al., 2004b). Thus, the efficient establishment of heterochromatin results in rapid homogenization of *h*^*90*^ cell populations to almost equal proportions of *P* and *M* cells, maximizing mating and meiosis and therefore spore formation. In *h*^*90*^ cells, the information present at *mat1* is chosen by biased mating-type switching between *mat2* or *mat3*, with ∼90% probability of selecting the opposite mating type (Klar, 1990). This strong bias selection depends on the heterochromatin state of the *mat* locus, so that cells with mutant Swi6, required to assemble heterochromatin, are defective in selecting donor choice and biases *h*^*90*^ cell populations toward the *mat1-M* (minus) owing to the preferred use of an adjacent recombination enhancer close to *mat3*. This enhancer is occupied by the Swi2-Swi5 recombination complex, required to promote mating-type switching; *h*^*90*^ cultures defective in Swi2 but with proper heterochromatin structure in the area have an accumulation of *mat1-P* (plus) cells, as *mat2* is properly positioned relative to *mat1* (Jia et al., 2004b).

Another interpretation of the role of heterochromatin in the process of directionality of switching comes from the group of Thon (Jakociunas et al., 2013), and can complement the model proposed by Jia and colleagues (Jia et al., 2004b). According to this new model, both genes at the *mat* locus, *mat2,* and *mat3*, have recombination enhancers capable of recruiting the Swi2-Swi5 recombination complex. Although the enhancer close to *mat3* has more affinity for the complex, *mat1* has a preferential choice for the cassette adjacent to *mat2*. This new model proposes that *M* cells display higher local abundance of Swi6 and Swi2; in this situation, both recombination sites would be bound by Swi2, and the position around *mat2* would be the preferred choice of recombination. In *P* cells, low concentration and association of Swi6 and Swi2 to the *mat* locus would promote invasion from *mat1* of the recombination site adjacent to *mat3*.

The *cis* and *trans* elements regulating heterochromatin assembly at the *mat* locus have been studied during the last decades. Many of the original identifications came from the selection of mutants with enhanced expression of a *ura4* transgene inserted in the silent *mat* locus (Ekwall and Ruusala, 1994; Thon et al., 1994; Thon and Klar, 1992). It was early reported the participation of histone deacetylases (HDACs) Clr6 and Clr1-Clr2-Clr3 (SHREC) complex (Kim et al., 2004; Yamada et al., 2005). Histone H3 and H4 sequential deacetylation by these HDACs mediates the methylation of H3 at lysine 9 (H3K9me) by Clr4, and recruitment of Swi6 to these modified histone marks (Ekwall and Ruusala, 1994; Thon et al., 1994; Thon and Klar, 1992). It was later discovered that heterochromatin assembly at *mat* locus involves the activity of small non-coding RNAs associated with the RNA interference (RNAi) pathway. Thus, several proteins of the RNAi pathway such as Dicer (Dcr1), RNA-dependent RNA polymerase (Rdp1), and Argonaute (Ago1) are essential for heterochromatin assembly at the *cis cenH* element [96% similar to *dg* and *dh* centromeric repeats (Grewal and Klar, 1997)] in the *mat2/3* region (Hall et al., 2002; Volpe et al., 2002). This pathway then promotes the recruitment of Clr4 and Swi6 (Grewal, 2000; Hall et al., 2002; Noma et al., 2004; Pidoux and Allshire, 2004; Volpe et al., 2002, 2003).

Once established the critical role of the RNAi pathway to initiate heterochromatin formation at the *mat* locus around *cenH*, it was soon highlighted that assembly still occurs in mutants of the RNAi pathway at lower efficiency. An unexpected connection between heterochromatin assembly at the *mat* locus and the stress-responsive transcription factor (TF) Atf1 was proposed by the groups of Grewal and Park (Jia et al., 2004a; Kim et al., 2004). Thus, in parallel to the *cenH-*RNAi-dependent pathway, the heterodimeric TF Atf1-Pcr1 regulates the establishment of heterochromatin at the *mat* locus by binding the heterodimer to two cAMP-response elements (*CRE*) sites adjacent to the *mat3* locus (Jia et al., 2004a; Kim et al., 2004). Its binding to the area targets the HDAC Clr3, to then contribute together with the RNAi cascade to the recruitment of the H3K9 methylase Clr4 and the H3K9me reader Swi6 to assemble and spread heterochromatin (Yamada et al., 2005); direct recruitment of Clr6 (Kim et al., 2004) or Clr4 (Wang and Moazed, 2017) by Atf1 to the *mat* locus has also been proposed. In conclusion, the Clr4-dependent methylation mark and heterochromatin assembly at the *mat* locus depend on two distinct mechanisms, involving the RNAi machinery, through *cenH*, and the site-specific DNA binding protein Atf1-Pcr1 heterodimeric TF (Jia et al., 2004a; Kim et al., 2004). It has recently been proposed that Atf1 participates in the epigenetic inheritance of the silent H3K9me mark at the *mat* locus, in combination with other transcription and replication factors, contributing to the spreading from the nucleation *cenH* site and to the high fidelity of the repressed state (Greenstein et al., 2018; Wang and Moazed, 2017; Wang et al., 2021).

The TF Atf1 was originally described as a signal-responsive factor mediating homologous recombination, sexual development, and a general anti-stress transcriptional program in response to signals (Kanoh et al., 1996; Takeda et al., 1995; Wahls and Smith, 1994). It works downstream of the MAP kinase Sty1 (Shiozaki and Russell, 1996; Wilkinson et al., 1996). The *S*. *pombe* Sty1/Spc1 MAP kinase is a general regulator of anti-stress responses. Environmental signals such as heat shock, nutritional starvation or osmotic or oxidative stress, compromising cell survival, activate Sty1, which then accumulates at the nucleus and phosphorylates the TF Atf1 to activate a gene expression program (Chen et al., 2008; Millar et al., 1995; Salat-Canela et al., 2017; Shiozaki and Russell, 1995, 1996; Wilkinson et al., 1996). Atf1 has also been described as a repressor at some genomic loci, before and after stress imposition (Degols and Russell, 1997; Sanso et al., 2008). The molecular bases of the effect of Atf1 phosphorylation vary depending on the process regulated and on the chromatin context. In response to hydrogen peroxide, more than 500 genes are up-regulated more than 2-fold, these changes greatly depending on Sty1 and Atf1 (Chen et al., 2003, 2008). Cells expressing a hypo-phosphorylation Atf1 mutant, Atf1.10M, lacking 10 out of 11 canonical MAP kinase phosphorylation sites (serine or threonine followed by proline), are sensitive to oxidative stress and cannot trigger the transcription of a group of genes including the catalase-coding *ctt1* (Salat-Canela et al., 2017). A subset of stress genes is still induced in a stress- and Sty1-dependent manner in these cells, which suggests that other non-canonical sites in Atf1 may be sufficient to promote these transcription events (Salat-Canela et al., 2017). Regarding the activation of these stress genes, Atf1 phosphorylation in 6 out of the 11 consensus sites, clustered in a central domain away from the DNA-binding domain according to modeling studies, is sufficient for transcriptional activation (Salat-Canela et al., 2017).

Sty1 and Atf1 have also been implicated in other cellular processes besides their essential role in the activation of stress genes. Thus, they are required to trigger homologous recombination at the mutated chromosomal locus *ade6-M26* (Wahls and Smith, 1994), they are involved in the initiation of mating and meiosis through the regulation of *ste11* transcript levels (Maeda et al., 1990; Mochizuki and Yamamoto, 1992; Sugimoto et al., 1991), and they participate in the response to glucose starvation by transcriptional up-regulation of the fructose-1,6-bisphosphatase coding gene (*fbp1*), a key enzyme in the gluconeogenic pathway (Hoffman and Winston, 1991). Canonical and non-canonical MAP kinase phospho-sites in Atf1 are required in all these processes, Sty1 being essential in all cases. Indeed, the expression of the phosphomimic Atf1.10D mutant can bypass the Sty1 requirement in the regulation of transcription and homologous recombination in all these events (Sanchez-Mir et al., 2020). In the regulation of most of these processes, Atf1 binds to its consensus *CRE* sites forming a heterodimer with the bZIP-containing TF Pcr1 (Janoo et al., 2001; Kanoh et al., 1996; Lawrence et al., 2007; Sanso et al., 2008; Wahls and Smith, 1994). The activity of the TF in most of these functions has been linked to the recruitment of chromatin-modifying complexes, such as SAGA (Adachi et al., 2018; Sanso et al., 2011; Yamada et al., 2004), Set1-COMPASS or the Paf1 complex (Garcia et al., 2016), or chromatin remodelers (Adachi et al., 2018; Hirota et al., 2008; Yamada et al., 2004), which normally trigger the local relaxation of the chromatin structure.

As explained above, non-activated Atf1 has been already linked to transcriptional repression, at least in the absence of stress, suggesting that the TF may have domains to recruit chromatin-compacting complexes as described for its role at the *mat* locus. However, why is this TF, capable of shifting from a repressor to an activator state in a signal- and Sty1-dependent manner, contributing to heterochromatin assembly at *mat?* Is this plasticity required for its function at the locus? We have tested whether the phosphorylation of the TF would have an impact on the establishment of the epigenetic marks that allow the assembly of heterochromatin at the *mat* locus by using Atf1 phospho mutants. We demonstrate that the phosphomimic Atf1.10D is unable to assemble heterochromatin at *mat*, while the hypo-phosphorylation mutant Atf1.10M is exceptionally suited to establish silencing at the locus. All the forms of Atf1 capable of establishing heterochromatin do so by mediating Clr3-containing SHREC complex recruitment to the *mat* locus under basal conditions. Importantly, expression of this stress-blinded Atf1.10M can alter the directionality of switching between the donor *mat2/3* and the acceptor *mat1* in less than 25 generations while preserving heterochromatin integrity, which demonstrates that both the repressive and activating functions of the TF are required for functionality at the *mat* locus.

## Results

### The phosphomimic Atf1.10D mutant is unable to promote chromatin silencing at the *mat* locus

**Figure 1:**
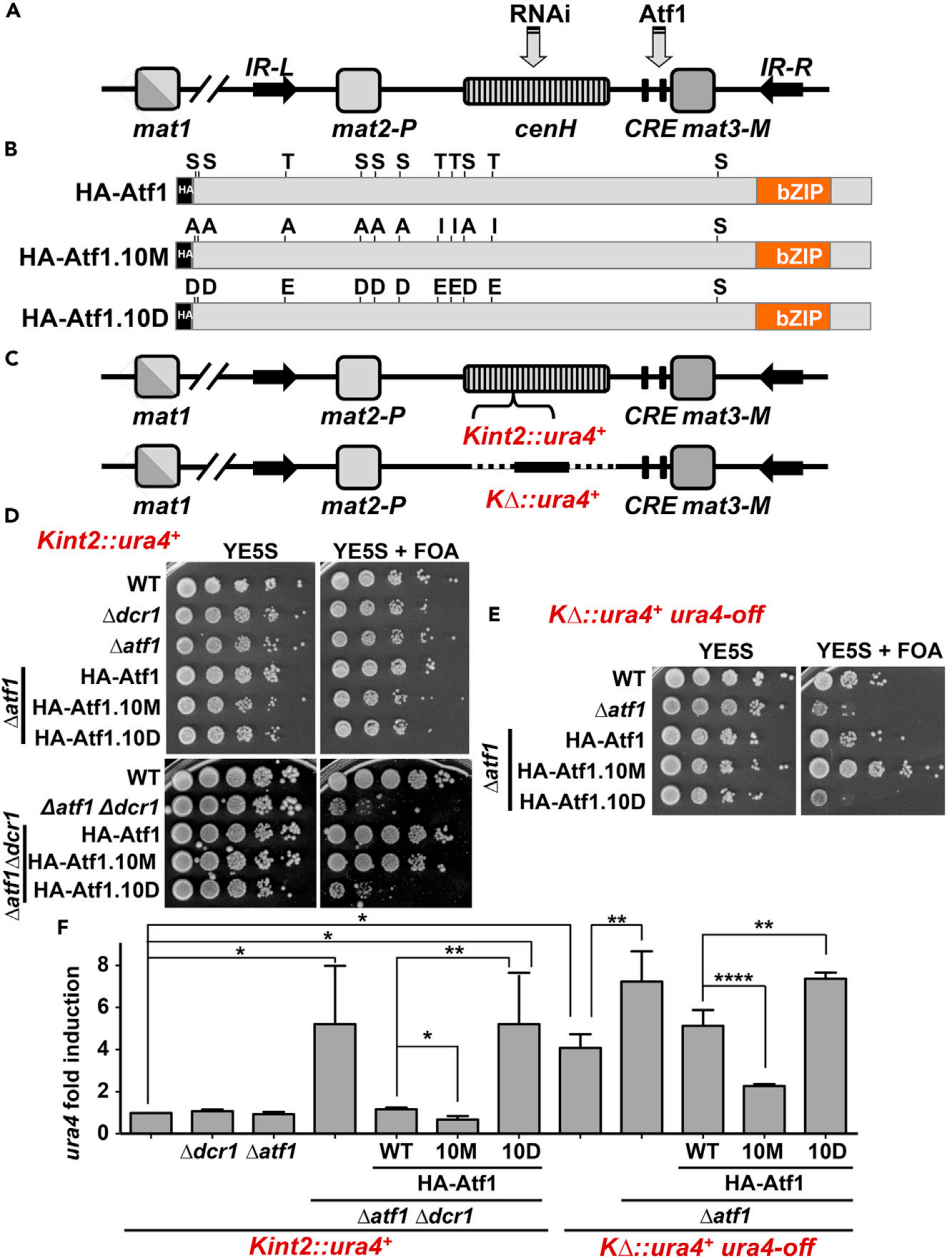
Atf1 phosphorylation affects heterochromatin establishment and maintenance at the *mat* locus (A) Schematic diagram of the *mat locus* of *S*. *pombe*, including the acceptor region (*mat1*), the donor cassettes (*mat2* and *mat3)*, the RNAi-dependent *cenH* element, the cAMP-response elements (*CRE*) sites, and the boundary sequences *IR-R* and *IR-L*. (B) Scheme of the Atf1 transcription factor, highlighting all the MAP kinase phosphorylation sites and their modifications to create a hypo-phosphorylated mutant (HA-Atf1.10M) and a phosphomimic one (HA-Atf1.10D), respectively. (C) Types of *mat* alleles. The *Kint2*::*ura4*^*+*^ system has a *ura4* reporter inserted in the *cenH* region. In the second system, *KΔ*::*ura4*^*+*^, the whole *cenH* is deleted and replaced with a *ura4* cassette. (D) Role of Atf1 and RNAi in heterochromatin at *Kint2*::*ura4*^*+*^. Serial dilutions of exponentially growing cultures of the indicated strains were spotted on YE5S plates with or without FOA (1 mg/mL). (E) Role of Atf1 in heterochromatin at *KΔ*::*ura4*^*+*^. The indicated strains were analyzed as in D. (F) Expression of *ura4* mRNA as an indicator of heterochromatin formation. *ura4* mRNA levels were analyzed by reverse transcriptase quantitative PCR (RT-qPCR). Total RNA from strains in D and E were obtained and quantified by RT-qPCR, as described in STAR Methods. Amplification with *act1* primers was used as a control for normalization. Data are presented as mean ± SD; ∗p < .05; ∗∗p < .01; ∗∗∗∗p < .0001 (Student’s t test). Each column represents the mean value and SD, calculated from at least three biological replicates. See also Figure S1.

The RNAi pathway and the Atf1-Pcr1 TF work in synergy to assemble heterochromatin at the *mat* locus, and their action is mediated by RNA generated from the *cenH* element or through binding to the *CRE* sites, respectively (Figure 1A). To determine whether stress- and Sty1-dependent phosphorylation of the TF affects its role at *mat*, we investigated the participation of Atf1 phosphorylation mutants, HA-Atf1.10M and HA-Atf1.10D (Figure 1B) in heterochromatin assembly at *mat* locus. We expressed wild-type Atf1 and mutant derivatives in homothallic *h*^*90*^ cells, containing a *ura4* cassette either inside the *cenH* region (*Kint*::*ura4*^*+*^) (Nakayama et al., 2001) or with the *ura4* cassette replacing the whole *cenH* region (*KΔ*::*ura4*^*+*^) (Grewal and Klar, 1997) (Figure 1C). Using these two strain backgrounds, it was demonstrated that the RNAi machinery cooperates with *cenH* to initiate heterochromatin assembly (Hall et al., 2002), and that in their absence heterochromatin assembly can still occur at low efficiency thanks to the Atf1-Pcr1 heterodimeric TF (Jia et al., 2004a; Kim et al., 2004).

In the *Kint*::*ura4*^*+*^ *h*^*90*^ strain, the absence of the RNAi machinery component Dcr1 or of the TF Atf1 does not have any impact on the silencing of the area: wild-type, *Δdcr1* or *Δatf1* cells can grow in 5-fluoroorotic acid (FOA)-containing plates and barely grow on plates without uracil, indicative of heterochromatin formation around the ectopic *ura4* cassette inserted in *mat*. On the contrary, cells lacking both Atf1 and Dcr1 cannot grow on FOA plates (Figure 1D) and grow in the absence of uracil (Figure S1) and therefore display silencing defects, as previously described (Jia et al., 2004a). The defects of *Δdcr1 Δatf1* cells were fully suppressed by expression from an integrative plasmid of wild-type Atf1 or Atf1.10M, while the phosphomimic Atf1.10D could not complement this strain (Figure 1D).

The *KΔ*::*ura4* strain, in which part of the *K* domain, including the RNAi-dependent *cenH* element, was replaced by a *ura4* marker (Grewal and Klar, 1996), could be isolated as a stably inherited *ura4-off* state (Grewal and Klar, 1997) (*KΔ*::*ura4*^*+*^ *ura4-off*; Figure 1E). This strain had an intermediate silencing phenotype, and it displayed some defects to grow on FOA plates which were exacerbated in the absence of Atf1 (Figure 1E); these differences in silencing could not be highlighted in plates lacking uracil, as all the backgrounds expressed sufficient levels of the *ura4* gene product (Figure S1). Again, only wild-type Atf1 and Atf1.10M, but not Atf1.10D, were able to suppress the defects of *Δatf1* on silencing, with Atf1.10M significantly improving the survival of a *KΔ*::*ura4*^*+*^ background (compare HA-Atf1 and HA-Atf1.10M in Figure 1E).

We performed quantitative PCR (qPCR) to confirm that the *ura4* mRNA levels justified the phenotypes observed on FOA plates. As shown in Figure 1F, the *ura4* transcript levels only raised in *Kint*::*ura4*^*+*^ *Δdcr1 Δatf1* cells in the absence of Atf1 or upon expression of the phosphomimic Atf1.10D, confirming the lack of silencing at the *mat* locus. Regarding the *KΔ*::*ura4*^*+*^ background, the *ura4* mRNA levels were quite elevated even in the absence of mutations, but almost doubled in cells lacking Atf1. Again, while Atf1.10D could not suppress the high *ura4* levels of *Δatf1* cells, Atf1.10M decreased to half the *ura4* transcripts of a *KΔ*::*ura4*^*+*^ background.

We conclude that a stress-blinded TF, Atf1.10M, is capable of triggering silencing more efficiently than wild-type Atf1, while Atf1.10D, mimicking a constitutively active TF, cannot contribute to heterochromatin assembly at the *mat* locus.

### The stress-sensing phosphorylation domain in Atf1 is required to promote heterochromatin assembly at *mat*

**Figure 2:**
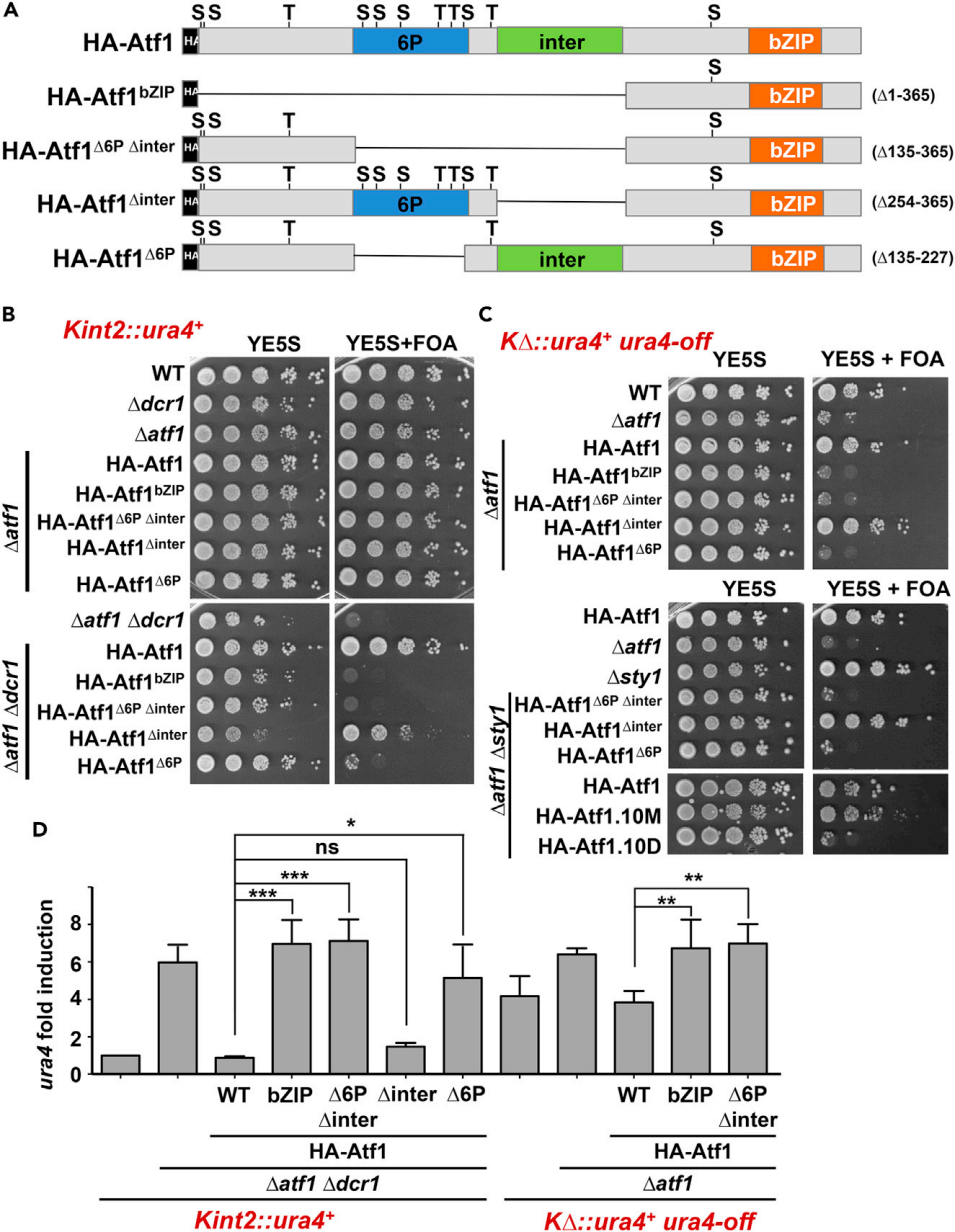
Deletion of the 6P domain in Atf1 blocks its heterochromatin assembly capacity at the *mat locus* (A) Scheme depicting the different truncated versions of Atf1. HA-Atf1.bZIP lacks the whole protein except the bZIP region. In HA-Atf1^Δ6P^, the domain containing 6 consensus phosphorylation sites required to induce transcription is deleted. HA-Atf1^Δinter^ has an intermediate region located downstream of the 6P domain deleted. Finally, HA-Atf1^Δ6P Δinter^ lacks 6P and inter domains. (B) Role of different Atf1 domains in heterochromatin at *Kint2*::*ura4*^*+*^. The indicated strains were analyzed as in Figure 1D. (C) Role of different Atf1 domains in heterochromatin at *KΔ*::*ura4*^*+*^. The indicated strains were analyzed as in Figure 1D. (D) Expression of *ura4* mRNA as an indicator of heterochromatin formation. RNA from strains in B and C were analyzed by RT-qPCR as described in Figure 1F. Data are presented as mean ± SD; ∗p < .05; ∗∗p < .01; ∗∗∗p < .001; ns, not significant (Student’s t test). Each column represents the mean value and SD, calculated from at least three biological replicates.

Even though further recruitment of Atf1 to some gene promoters has been reported after stress, the TF is already pre-bound to most of them prior to stress (Eshaghi et al., 2010; Salat-Canela et al., 2017). In fact, Atf1 can act as a repressor of stress genes prior to signaling and as an activator after environmental perturbations, with phosphorylation by Sty1 being essential to trigger this shift (Degols and Russell, 1997; Sanso et al., 2008). During our previous characterization of the role of the Sty1-dependent phospho-sites in Atf1 on transcription, we concluded that the phosphorylation of 6 out of the 11 serine or threonine followed by proline consensus sites, located in the domain indicated as 6P in Figure 2A, was sufficient for transcriptional activation (Salat-Canela et al., 2017), suggesting that, once phosphorylated, the 6P domain recruits transcription promoting complexes such as the histone acetyltransferase Gcn5-containing SAGA complex or RNA polymerase II machinery components (Sanso et al., 2011). To determine which domains in Atf1 are required for silencing at *mat*, we expressed in *Kint*::*ura4*^*+*^ and *KΔ*::*ura4*^*+*^ cells truncated versions of the TF, such as only its DNA binding domain (bZIP in Figure 2A) or lacking the 6P or an intermediate domain (inter) located between the phosphorylation and the bZIP domains (Figure 2A).

Expression of Atf1^bZIP^, containing only the DNA binding domain of Atf1, was not sufficient to generate silencing *in Kint*::*ura4*^*+*^ *Δdcr1 Δatf1* cells (Figure 2B), suggesting that this truncated version is not capable of recruiting the silencing complexes required to close chromatin in this region. The same occurs with Atf1^Δ6P Δinter^ and Atf1^Δ6P^, while Atf1^Δinter^ was fully capable of establishing silencing and promoting growth on FOA plates (Figure 2B). These results are recapitulated in *KΔ*::*ura4*^*+*^ *Δatf1* cells (Figure 2C, upper panels). Importantly, the effect on silencing upon expression from a constitutive promoter of the Atf1 phospho mutants 10M and 10D or of the Atf1 truncated derivatives in *KΔ*::*ura4*^*+*^ cells was not dependent on Sty1 (Figure 2C, bottom panels), suggesting that the 6P domain of Atf1 is capable of recruiting repressive complexes in a signal- and kinase-independent fashion.

Again, the expression of *ura4* transcripts from the *mat* locus fully paralleled the phenotypes of the truncated Atf1 proteins, with high expression levels correlating with low viability on FOA plates (Figure 2D). The analysis of these Atf1 derivatives suggests that the 6P domain of Atf1 is required to assemble heterochromatin at the *mat* locus.

### Atf1 bound to the minimal *KΔ*::*ura4*^*+*^*mat* locus can recruit the SHREC complex through the 6P domain

**Figure 3:**
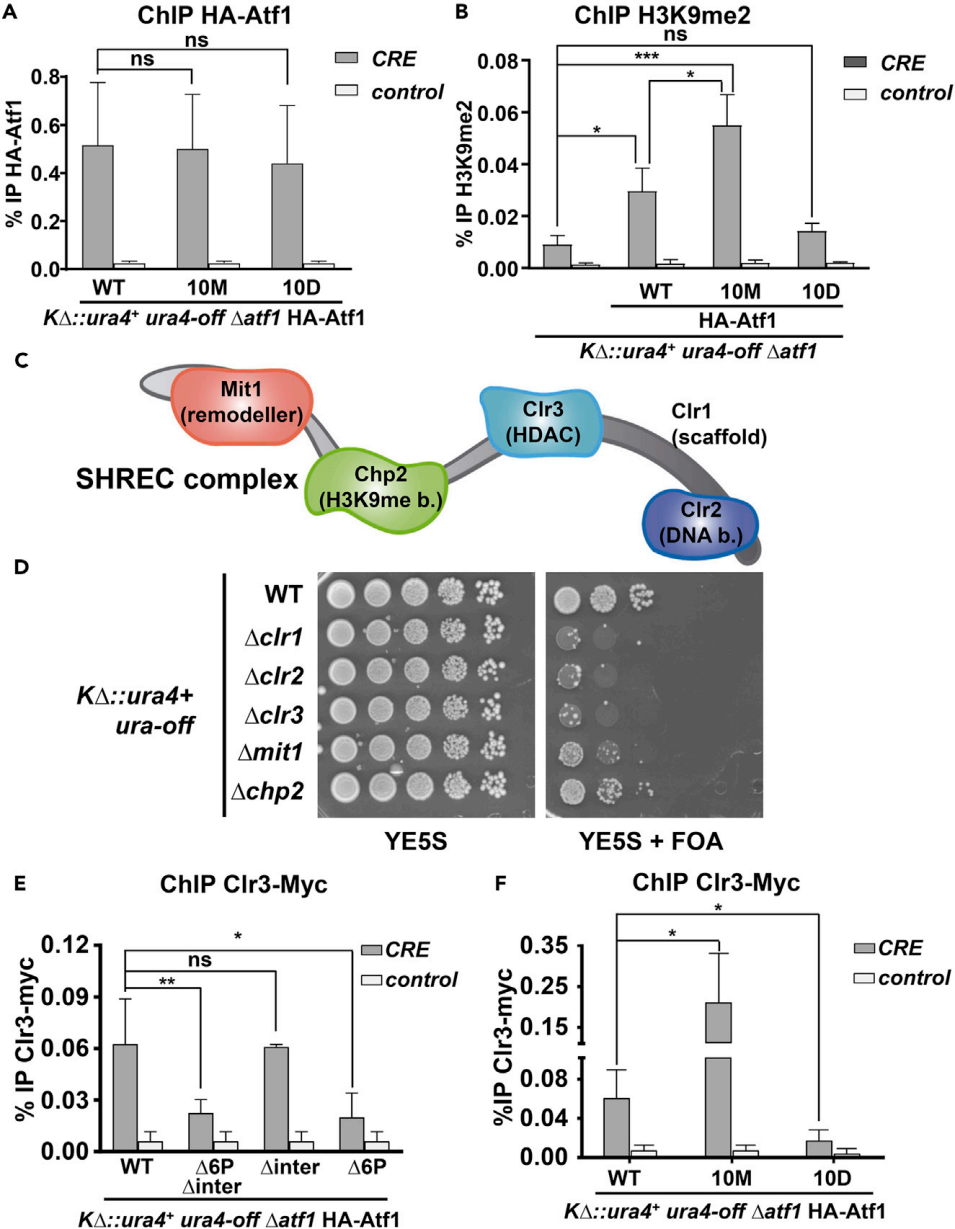
The SHREC complex is recruited by Atf1 to the *mat locus*, and is essential to silence heterochromatin (A) Atf1 and its mutant derivatives 10M and 10D bind to the *CRE* site at the *mat* locus. Extracts of YE cultures of the indicated strains were analyzed by ChIP using anti-HA, coupled with quantification by real-time PCR with primers covering the *CRE* site and the *act1* ORF as a negative control. Data are presented as mean ± SD obtained from biological triplicates; ns, not significant (Student’s t test). (B) Expression of Atf1 and Atf1.10M favors the deposition of the heterochromatin H3K9me2 mark at *mat*. Strains used were SPJ236 (*h*^*90*^*KΔ*::*ura4*^*+*^*ura4-off Δatf1*) and strains as in A. ChIP experiments were performed as in A using anti-H3K9me2 antibodies; for quantification, primers covering the *CRE* site and the mitochondrial DNA as a negative control were used. Data are presented as mean ± SD obtained from biological triplicates; ∗p < .05; ∗∗∗p < .001; ns, not significant (Student’s t test). (C) Scheme depicting the modular organization of the SHREC complex, with its five subunits. (D) Role of SHREC in heterochromatin at *KΔ*::*ura4*^*+*^. The indicated strains were analyzed as in Figure 1D. (E) Atf1 recruits SHREC to the *CRE* site at *mat* through the 6P domain. The indicated strains were analyzed by ChIP experiments performed as in A using anti-Myc antibodies; for quantification, primers covering the *CRE* site and the *act1* ORF as a negative control were used. Data are presented as mean ± SD obtained from biological triplicates; ∗p < .05; ∗∗p < .01; ns, not significant (Student’s t test). (F) Atf1 and Atf1.10M, but not 10D, recruits SHREC to the *CRE* site at *mat*. The indicated strains were analyzed by ChIP experiments performed as in A using anti-Myc antibodies; for quantification, primers covering the *CRE* site and mitochondrial DNA as a negative control were used. Data are presented as mean ± SD obtained from biological triplicates; ∗p < .05 (Student’s t test). See also Figure S2.

Binding of Atf1 to the *CRE* site at the *mat* locus was not affected by the presence or not of its phospho-sites, as shown by chromatin immunoprecipitation (ChIP) in Figure 3A. Nevertheless, only cells expressing wild-type and Atf1.10M, but not Atf1.10D, displayed high levels of the heterochromatin mark H3K9me2 (Figure 3B), suggesting that Atf1 contributes to silencing through the regulation of the histone code at the *mat* locus.

Several repressive complexes have been linked to the role of Atf1 in *mat* silencing, with the group of Grewal proposing the participation of the HDAC Clr3-containing complex, or SHREC (Yamada et al., 2005). Thus, the silencing defects observed in cells lacking Atf1 would be caused by the increase in histone H3 acetylation at the *mat* locus, which would subsequently affect the H3 methylation pattern (Yamada et al., 2005). The SHREC complex, as proposed by the groups of Schalch and Partridge, is composed of two sub-complexes, which are connected by the scaffolding flexible protein Clr1. The first sub-complex is composed of the chromatin remodeler Mit1 and the H3K9me-binding protein Chp2, while the HDAC module includes the HDAC Clr3 and the Clr2 protein, which also has a domain capable of recognizing heterochromatin marks (Job et al., 2016) (Figure 3C). We investigated whether both submodules of the SHREC complex were required for heterochromatin assembly at the minimal *mat* locus of the strain *KΔ*::*ura4*^*+*^. As shown in Figures 3D and S2, Clr1, Clr2, Clr3, and Mit1, but not the chromatin-binding protein Chp2, were required to silence the *mat* locus in the absence of *cenH*.

As shown again with ChIP using strains expressing Clr3-Myc and truncated versions of Atf1, the TF was capable of promoting Clr3 recruitment to the minimal *mat* locus around the *CRE* site, but not in the absence of the 6P domain (Figure 3E). Similarly, Atf1.10D could not recruit Clr3 to *mat*, while Atf1.10M did so with more efficiency than wild-type Atf1 (Figure 3F). We conclude that the same domain in Atf1, 6P in Figure 2A, can recruit both repressive (SHREC) and activating complexes, shifting from one to the other upon signal-dependent phosphorylation.

### Atf1.10M can fully restore mating efficiency of cells carrying an RNAi-independent minimal *mat* locus by enhancing heterochromatin and promoting bias switching

**Figure 4:**
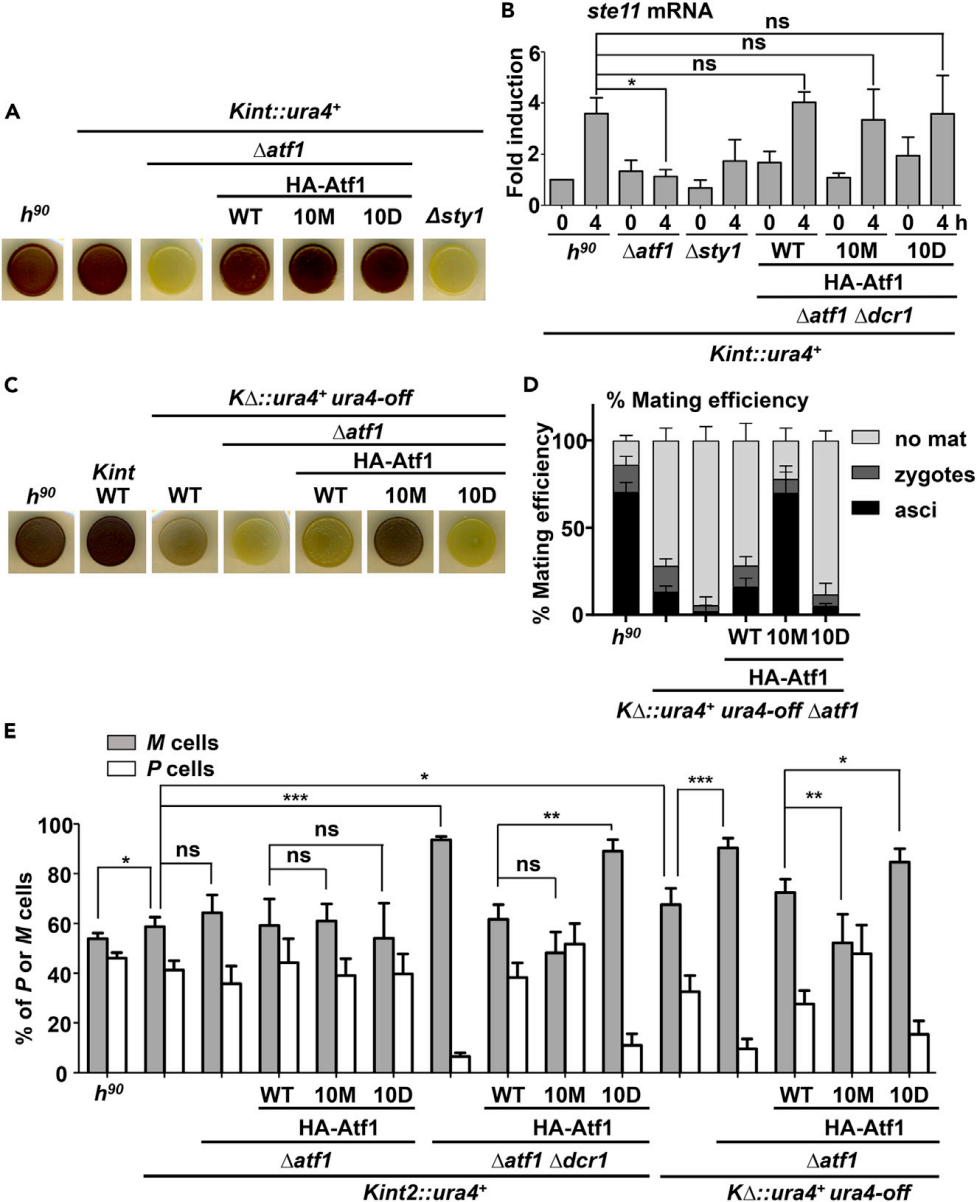
The hypo-phosphorylation mutant Atf1.10M rescues heterochromatin assembly at the minimal *KΔ*::*ura4*^*+*^ locus (A) Analysis of sporulation efficiency by iodine staining. Cells of the indicated strains were directly collected from strikes in rich media plates, resuspended in MM without nitrogen, and 10^5^ cells of each strain were dropped on sporulation plates. After three days growing at 25°C, plates were stained with iodine vapors and photographed. (B) Expression of the Sty1- and Atf1-dependent *ste11* transcript upon nitrogen depletion. Samples from the indicated strains were taken during exponential growth and 4 hours after switching to low-nitrogen media. RNA was analyzed by RT-qPCR as described in Figure 1F. Data are presented as mean ± SD; ∗p < .05; ns, not significant (Student's t test). Each column represents the mean value and SD, calculated from at least three biological replicates. (C) Analysis of sporulation efficiency by iodine staining of the indicated strains was performed as in A. (D) Mating efficiency rates (%) of the indicated strains were calculated as explained in STAR Methods from triplicates. The numbers of vegetative cells (light gray), zygotes (dark gray), or asci (black) are shown as stacked columns ± SD. Thus, each column corresponds to the total percentage of cells counted. (E) Analysis of directionality of switching by qPCR. Genomic DNA from the indicated strains was extracted from cultures grown on solid YE5S plates, and analyzed by qPCR using two sets of primers, one specific for *M* and the other for *P* cells. Data are presented as mean ± SD; ∗p < .05; ∗∗p < .01; ∗∗∗p < .001; ∗∗∗∗p < .0001; ns, not significant (Student’s t test), calculated from at least three biological replicates.

As explained in the Introduction, mutations in *swi6* or other genes required to establish heterochromatin at the *mat* locus reduce the efficiency of mating-type switching in *h*^*90*^ cells, as heterochromatin imposes a structural organization of the *mat* locus that allows biased donor choice during switching (Jia et al., 2004b; Thon and Klar, 1993). Thus, wild-type *h*^*90*^ cells grown on sporulation medium are darkly stained upon exposure to iodine vapors, owing to their high spore content, whereas colonies of *h*^*90*^ carrying a *swi6* mutation are lightly stained with iodine vapors (Jia et al., 2004b). Many other mutants not related to heterochromatin assembly or to *mat* switching can also yield *h*^*90*^ populations defective in sporulation. That is the case of *Δsty1* or *Δatf1 h*^*90*^ cells: after nitrogen depletion, activated Sty1 promotes the transcription of the *ste11* gene in an Atf1-dependent manner (Davidson et al., 2004; Kanoh et al., 1996; Shiozaki and Russell, 1996; Takeda et al., 1995); the Ste11 TF is essential to trigger the mating and meiosis program (Sugimoto et al., 1991). As shown in Figure 4A, when *h*^*90*^ *Kint*::*ura4*^*+*^ cells grown on sporulation media were exposed to iodine vapors, staining was very similar to that of wild-type *h*^*90*^ cells, indicating that spore formation is not impaired by the insertion of the *ura4* cassette. However, the deletion of the *atf1* or the *sty1* gene was sufficient to render non-mating populations which were not stained with iodine vapors. Expression of either wild-type Atf1 or its mutant derivatives Atf1.10M or 10D was sufficient to suppress the defects of cells lacking Atf1 (Figure 4A). As shown before in an *h*^*90*^ background (Sanchez-Mir et al., 2020), both phospho mutants were able to trigger the activation of *ste11* transcripts on nitrogen depletion (Figure 4B).

As shown in Figure 4C and reported before (Grewal and Klar, 1997), the *KΔ*::*ura4*^*+*^ background stained lighter than wild-type *h*^*90*^ cells with iodine vapors, owing to weaker heterochromatin formation and impaired directionality of switching, so that cells were partially defective in the utilization of the *mat2* (*P*, plus) cassette as a donor to *mat1* (Figure 4C). In this RNAi-independent minimal *mat* locus, the role of Atf1 and its mutant derivatives would be, therefore, crucial not only to promote the entry into the mating and meiosis program through the activation of *ste11* but also to assemble heterochromatin and promote biased switching. We freshly transformed again the *KΔ*::*ura4*^*+*^ *Δatf1* background with plasmids encoding Atf1 and mutant derivatives, and assayed their sporulation capacity by iodine staining. As expected, not only cells lacking Atf1 but also those expressing the phosphomimic Atf1.10D were non-mating and did not stain at all with iodine (Figure 4C). On the contrary, Atf1.10M seemed to be proficient to improve the sporulation of the *KΔ*::*ura4*^*+*^ background, as determined by the darker color of the colonies (Figure 4C). We confirmed these findings by measuring mating efficiently of wild-type *h*^*90*^ and of all our *KΔ*::*ura4+* derivatives: to our surprise, Atf1.10M can fully restore the mating efficiently of the *KΔ*::*ura4*^*+*^ to the levels of wild-type *h*^*90*^ cells (Figure 4D).

To confirm that the mating efficiencies of these *h*^*90*^ *KΔ*::*ura4*^*+*^ populations are directly connected to the proportion of *M* (minus) and *P* (plus) cells, we developed a qPCR-based assay to measure the genetic content at *mat1* from small cell populations, using genomic DNA isolated from strikes of recently transformed strains. We designed two pairs of primers, with a common one flanking the *mat1* locus and another specific for either *mat2* or *mat3*, and their precise efficiency was determined using equal amounts of genomic DNA from *h*^−^ and *h*^*+*^ cells within the same PCR reaction, and used these values to normalize quantifications from genomic DNA of *h*^*90*^ populations. As expected, wild-type *h*^*90*^ cells presented an almost equal number of *M* and *P* cells (54 and 46%, respectively), while the *h*^*90*^ *Kint2*::*ura4*^*+*^ background already displayed some divergence (58 and 42% of *M/P*), which was not largely affected by the deletion of *atf1* (64 and 36% of *M/P*)) (Figure 4E). However, the *M/P* ratio was largely shifted in the *Kint2*::*ura4* ^+^ *Δatf1 Δdcr1* background (93 and 7% of *M/P*), and this cell proportion was not restored by the expression of Atf1.10D, while Atf1.10M improved the ratio back to the wild-type *h*^*90*^ ratio (48 and 52% of *M/P*). Cell populations of the *h*^*90*^ *KΔ*::*ura4*^*+*^ background had a significant shift in the *M/P* ratio (68 and 32% of *M/P*), which explains their intermediate staining with iodine vapors and their low mating efficiency (Figures 4C and 4D). Populations of *KΔ*::*ura4*^*+*^ *Δatf1* displayed an exacerbated biased ratio (90 and 10% of *M/P*) (Figure 4E), confirming that this low proportion of *P* cells gives a phenotype of sterility; expression of Atf1.10D could not suppress this abnormal ratio nor the low mating efficiency (Figures 4C and 4D). On the contrary, Atf1.10M restored the *M/P* ratio back to the levels of a wild-type *h*^*90*^ population ratio (52 and 48% of *M/P*) (Figure 4E).

### In the minimal *KΔ*::*ura4*^*+*^*mat* locus, Atf1.10M can evolve from a potent heterochromatin assembly factor to a repressor of *mat2/3* switching

**Figure 5:**
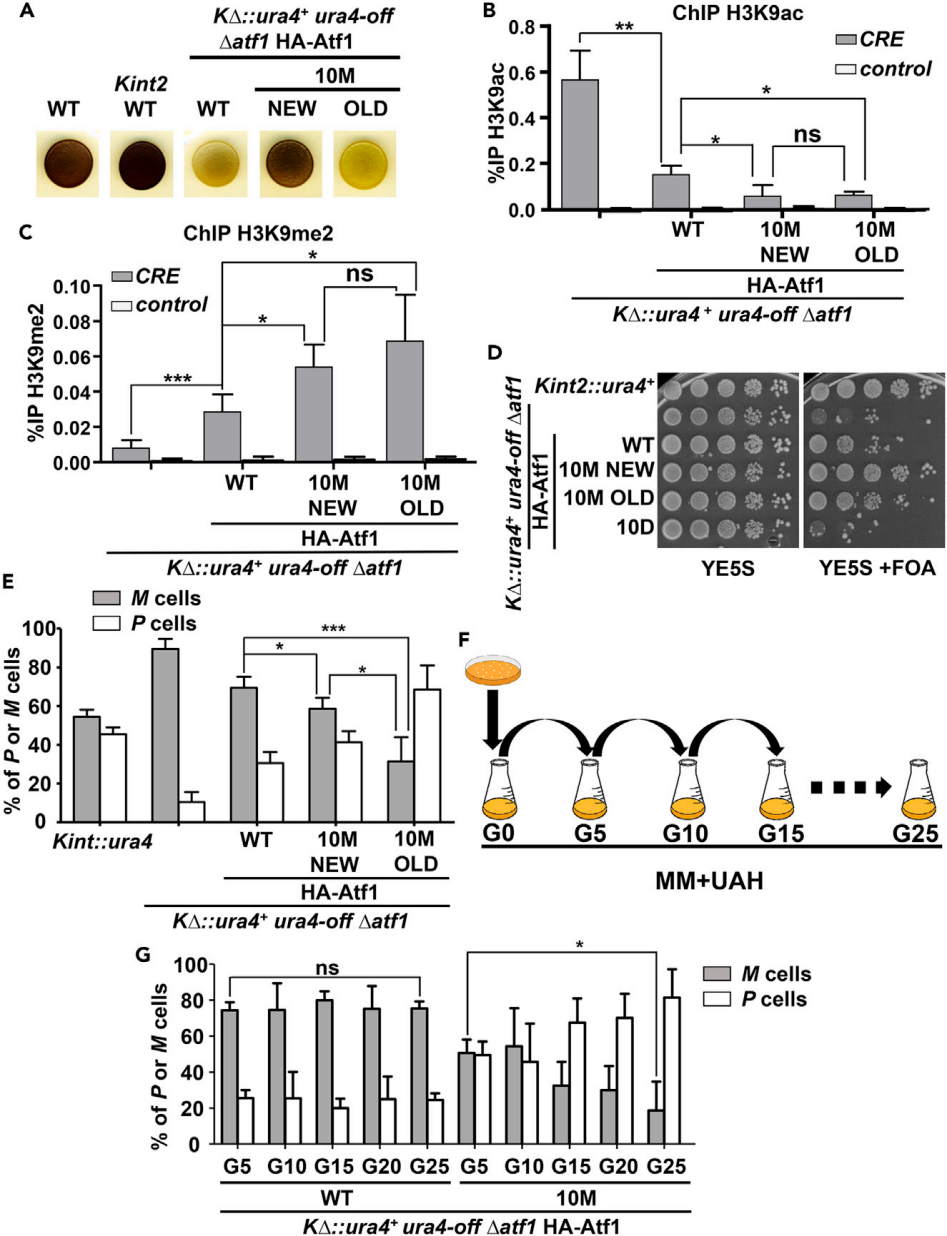
At the minimal *mat* locus, Atf1.10M can initially suppress the mating defects of cells lacking Atf1, but it evolves into a repressor of *mat2/3* switching and cells lose mating capacity (A) Analysis of sporulation efficiency by iodine staining of new and evolved HA-Atf1.10M-expressing strains. Analysis of sporulation efficiency by iodine staining was performed as in Figure 4A, from strain LS48.10M (*h*^*90*^*KΔ*::*ura4*^*+*^*ura4-off Δatf1* + HA-Atf1.10M), which has passed through several cultures passes (OLD) with a culture of a HA-Atf1.10M-freshly transformed strain (NEW). Control strains used were JA209 (*h*^*90*^), SPJ83 (*h*^*90*^*Kint2*::*ura4*^*+*^), SPG1161 (*h*^*90*^*KΔ*::*ura4*^*+*^*ura4-off*) and SPJ236 (*h*^*90*^*KΔ*::*ura4*^*+*^*ura4-off Δatf1*). (B) Deposition of the euchromatin mark H3K9Ac at *mat* is absent in both new and evolved HA-Atf1.10M-expressing strains. Strains used were SPJ236 (*h*^*90*^*KΔ*::*ura4*^*+*^*ura4-off Δatf1*), LS48 (*h*^*90*^*KΔ*::*ura4*^*+*^*ura4-off Δatf1* + HA-Atf1), and the two versions of LS48.10M (*h*^*90*^*KΔ*::*ura4*^*+*^*ura4-off Δatf1* + HA-Atf1.10M), OLD and NEW. ChIP experiments were performed as in Figure 3A using anti-H3K9ac antibodies; for quantification, primers covering the *CRE* site and a mitochondrial DNA region as a negative control were used. Data are presented as mean ± SD from biological triplicates; ∗p < .05; ∗∗p < .01; ns, not significant. (C) Deposition of the heterochromatin mark H3K9me2 at *mat* is promoted by both new and evolved HA-Atf1.10M-expressing strains. Strains and ChIP experiment were performed as in B, using anti-H3K9me2 antibodies. Data are presented as mean ± SD from four biological replicates; ∗p < .05; ∗∗∗p < .001; ns, not significant. (D) Both new and evolved HA-Atf1.10M-expressing strains can trigger heterochromatin assembly at *KΔ*::*ura4*^*+*^. Heterochromatin assembly capacity of strains as in B was analyzed on FOA plates as described in Figure 1D. As control strains, we used SPJ83 (*h*^*90*^*Kint2*::*ura4*^*+*^), SPJ236 (*h*^*90*^*KΔ*::*ura4*^*+*^*ura4-off Δatf1*), LS48 (*h*^*90*^*KΔ*::*ura4*^*+*^*ura4-off Δatf1* + HA-Atf1) and LS48.10D (*h*^*90*^*KΔ*::*ura4*^*+*^*ura4-off Δatf1* + HA-Atf1.10D). (E) The ratio of *P* and *M* cells changes dramatically in new and evolved HA-Atf1.10M-expressing strains. Analysis of directionality of switching by qPCR was performed as described in Figure 4E. We used strains as in B, with the control strain SPJ83 (*h*^*90*^*Kint2*::*ura4*^*+*^). Data are presented as mean ± SD; ∗p < .05; ∗∗∗p < .001 (Student’s t test), calculated from four biological replicates. (F) Scheme depicting the process of evolution followed in Figure 5G. A culture of strain SPJ236 (*h*^*90*^*KΔ*::*ura4*^*+*^*ura4-off Δatf1*) was transformed with HA-Atf1 or HA-Atf1.10M and plated in MM with supplements for selection. Positive colonies were inoculated into liquid cultures of MM with supplements, and every 5 generations the cultures were refreshed back to the starting point and samples were taken. Finally, genomic DNA was obtained from each sample and analyzed for *mat* switching. (G) Analysis of directionality of switching by qPCR of HA-Atf1 and HA-Atf1.10M populations, recently transformed or evolved. Genomic DNA was extracted from samples obtained as described in F, and the ratio of *P* and *M* cells was analyzed as described in Figure 4E. Data are presented as mean ± SD; ∗p < .05; ns, not significant (Student’s t test), calculated from biological triplicates. See also Figures S3 and S4.

As demonstrated by Jia, Grewal, and colleagues, heterochromatin at *mat* imposes the structural organization of the area that is required for the donor-choice switching mechanism, that is, positions the *mat2* donor locus in the proper location relative to the acceptor *mat1* (Jia et al., 2004b). The Swi2 recombination complex that accumulates close to *mat3* requires, however, some accessibility to dsDNA around *mat1*. With the idea that an excessive and constitutive silencing at *mat* locus in *KΔ*::*ura4*^*+*^ by Atf1.10M could lead to a blockage of recombination activities required for switching, we closely monitored the evolution of populations of *KΔ*::*ura4*^*+*^ *Δatf1* freshly transformed with Atf1.10M, and determined whether spore formation, heterochromatin assembly around the domain and the ratio of *M/P* cells in these populations were constant or evolved. As shown in Figure 4C and in Figure 5A with iodine vapors, colonies of *KΔ*::*ura4*^*+*^ *Δatf1* freshly transformed with Atf1.10M were prone to engage in the mating and meiosis program when spread on sporulation media, and in fact were as proficient as wild-type or *Kint*::*ura4*^*+*^ *h*^*90*^ cells (“NEW” in Figure 5A). However, we realized that this strain did not properly maintain its mating capacity, because after several passes on plates, the staining with iodine vapors of the adapted cell populations was clearly diminished (“OLD” in Figure 5A), to levels similar to *KΔ*::*ura4*^*+*^ cells. We ruled out that this strain, “OLD” in Figure 5A, displayed major chromosomal re-arrangements at the *mat* locus (Figure S3). To test whether the lack of mating efficiency of this “evolved” *KΔ*::*ura4*^*+*^ *Δatf1 atf1*.*10M* strain was owing to a decrease in heterochromatin levels at the domain, we first determined that the euchromatin histone mark H3K9ac was as low as in a recently transformed background (Figure 5B), and the heterochromatin mark H3K9me2 was as equally elevated in both the freshly transformed and evolved *KΔ*::*ura4*^*+*^ *Δatf1 atf1*.*10M* backgrounds (Figure 5C). Similarly, the *ura4* mRNA levels were not significantly elevated in the OLD 10M strain than the NEW 10M strain (Figure 5D). We then tested whether the lack of mating efficiency of this evolved strain was owing to a loss in biased switching. As shown in Figure 5E, the OLD 10M strain had lost the proper *M/P* ratio, with *P* (plus) cells accumulating (30 and 70% of *M/P*). Therefore, this shift in mating efficiency of the evolved (OLD) *KΔ*::*ura4*^*+*^ *Δatf1 atf1*.*10M* strain was not caused by a decrease in heterochromatin assembly capacity, which was maximal and constant, but rather by the accumulation of *P* cells.

To determine whether few generations of *KΔ*::*ura4*^*+*^ *Δatf1 atf1*.*10M* were sufficient to cause the change in the *M/P* ratio detected for the OLD versus NEW backgrounds, we systematically re-streaked freshly transformed colonies into minimal media-containing flasks, as indicated in Figure 5F, and determined the ratio of *M* vs. *P* cells in the different populations. As a control, we also freshly transformed the *KΔ*::*ura4*^*+*^ *Δatf1* strain with wild-type Atf1. Although this strain maintained the ratio of *M/P* cells at 72% and 28%, respectively, the populations from the Atf1.10M transformation did not: we detected a shift from 51 to 49% ratio of *M/P* cells in the starting populations (G5 in Figure 5G) to an 18 to 82% *M/P* ratio in evolved populations (G25 in Figure 5G). This accumulation of *P* cells is characteristic of strains with defects in the recombination complex such as *Δswi2* (Jia et al., 2004b). In conclusion, the minimal *mat* locus at *KΔ*::*ura4*^*+*^ can fully assemble heterochromatin with the aid of the stress-blinded Atf1.10M TF, but the lack of signal- and kinase-dependent plasticity of this mutant Atf1 impairs the normal donor-choice switch, favoring the selection of *mat2* as a donor to *mat1*.

## Discussion

In fission yeast, heterochromatin assembly is a requisite for proper switching of the genetic information contained at *mat1* using as templates the donors *mat2* or *mat3* in *h*^*90*^ populations, so that this switch is biased and the number of *P* and *M* cells remains almost equal, and mating and meiosis can be efficient upon nutritional deprivation. Heterochromatin assembly at *mat* locus is synergistically ruled by two independent pathways, and at least one of them can be modulated by environmental signals and even during a physiological cell cycle: cells lacking the Sty1 kinase display strong phenotypic defects during stress imposition, but they also have an elongated phenotype such as other cell cycle division mutants (Salat-Canela et al., 2021). We propose that the regulation of Atf1 activity by the MAP kinase cascade may be required to establish a dynamic chromatin structure favorable to both silencing but also suitable to allow the switching process at the mating-type region during an unperturbed cell cycle.

To analyze the putative role of active *vs*. inactive Atf1 in *mat* assembly, we have constitutively expressed phospho mutants and truncated versions of the TF in different genetic backgrounds, instead of characterizing *Δsty1* cells, as they display pleiotropic defects. When the role of Atf1 at *mat* was originally studied by the group of Park, silencing at the *mat* locus was comparable in wild-type and *Δsty1* cells, but the stabilization of the epigenetic inheritance of *S*. *pombe* cells, partially disrupted in cells lacking Atf1, was enhanced in cells lacking the upstream Atf1 kinase Sty1 (Kim et al., 2004). This already suggested that the loss of Sty1 might strengthen the repressive/silencing activity of Atf1, even under unstressed conditions. It is worth pointing out, however, that cells lacking Sty1 display almost undetectable levels of Atf1, as *atf1* transcript levels depend on phosphorylated Atf1 (Salat-Canela et al., 2017). Therefore, the use of constitutively expressed Atf1 derivatives has allowed us to unambiguously conclude that a stress-blinded TF, Atf1.10M, is capable of triggering silencing more efficiently than wild-type Atf1. Indeed, when expressed in *KΔ*::*ura4*^*+*^ cells, with a minimal *mat* locus displaying the intermediate capacity to silence the domain and to promote mating and meiosis, Atf1.10M can fully restore heterochromatin assembly capacity to overcome the lack of the *cenH-*RNAi system.

Atf1 is a potent transcriptional activator (Chen et al., 2003), but its role as a repressor of transcription or recombination has also been demonstrated (Degols and Russell, 1997; Eshaghi et al., 2010; Gao et al., 2008; Sanso et al., 2008). In fact, the group of Wahls proposed that two distinct domains in the TF are necessary and sufficient to activate and repress homologous recombination at the *ade6*.*M26* locus (Gao et al., 2008). Our study demonstrates that the 6P domain, including six Sty1-dependent phosphorylation sites which are essential and sufficient for the transcriptional activation of stress genes, is also required for the silencing capacity of the TF. We propose that, when non-phosphorylated, Atf1 recruits the SHREC complex towards the *mat* locus; on the contrary, signal- and Sty1-dependent phosphorylation of these phospho-sites may trigger a shift in binding partners by the TF, as transcription-promoting complexes such as SAGA and RNA polymerase II itself have been described to be recruited by active Atf1 (Sanso et al., 2011). Although in an intact *mat* locus the SHREC complex was originally proposed to be recruited by histone modifications initiated at the *cenH* region, Grewal and colleagues proposed that the initial recruitment of Clr3 is performed by Atf1, and its spreading is severely defective in a *swi6* mutant (18). The role of Atf1 in SHREC recruitment is confirmed here with the use of the minimal *mat* locus at *KΔ*::*ura4*^*+*^, with the subunit recognizing heterochromatic histone marks, Chp2, being dispensable for the role of SHREC in promoting silencing. That is, in the absence of the main heterochromatin nucleation center, *cenH*, Atf1 rules the direct recruitment of at least the Clr3-containing SHREC histone deacetylase complex, generating the establishment of histone deacetylation patterns at the locus, which will then be followed by other histone modifications such as methylation.

Why are there two systems, RNAi and Atf1, required to establish heterochromatin at *mat*, if a stress-blinded DNA binding protein such as Atf1.10M, capable of recruiting a chromatin deacetylase complex, would be sufficient? And what is the biological relevance of Atf1 interconversion between an activator and a repressor? Regarding chromatin architecture at the *mat* locus, silencing of the domain is not the only process required to promote mating and meiosis. Thus, *swi6* mutant cells, with defects in heterochromatin establishment, display *h*^*90*^ populations predominantly of the *mat1-M* (*M*, minus) mating type, as the lack of a rigid heterochromatin architecture disfavors recombination between *mat1* and *mat2*, whereas cells defective in the recombination complex component Swi2 accumulate mainly with the *mat1-P* (*P*, plus) mating type, since established heterochromatin puts in close proximity and permits recombination between *mat1* and *mat2*; both *swi6* mutant and *Δswi2* stain lightly with iodine (Jia et al., 2004b). Similarly, cells lacking Atf1 or expressing Atf1.10D display silencing defects and were non-mating owing to the accumulation of *M* cells, mimicking a *swi6* mutant phenotype, as they are unable to assemble heterochromatin. On the contrary, Atf1.10M, which was even capable of transforming the minimal *mat* locus of the *KΔ*::*ura4*^*+*^ background into a strong silencing nucleation center, became not capable of mating after few cell divisions owing to the accumulation of *P* cells, resembling the phenotype of cells lacking Swi2. By interacting through the same domain with complexes that open and close chromatin, Atf1 may provide an interface capable of promoting heterochromatin silencing but transiently allowing mating-type interconversion, favorable to both apparently antagonistic processes. Another simple interpretation of our results, based on the Jakociunas model described in the introduction (Jakociunas et al., 2013), is that the increase of heterochromatin levels induced by Atf1.10M in the mating-type region causes a progressive excess of Swi2-Swi5 in the area, to a state that is ultimately similar to that normally present in *M* cells, leading to preferential use of *mat2*. In both the Jia et al. (2004b) and the Jakociunas et al. (2013) models, high levels of heterochromatin favor the use of *mat2* during switching. Of note, *h*^*+*^ and *h*^*-*^ cells do not display differences in the levels of phosphorylation of Atf1 (Figure S4). In conclusion, we propose that the regulation of wild-type Atf1 activity by the MAP kinase cascade may be required to establish this dynamic chromatin structure favorable to both silencing and switching processes at *mat*. Whether Atf1 recruits directly the Swi2-recombination complex or opens the chromatin through SAGA to allow the recruitment of the former is under investigation.

## Limitations of the study

In order to analyze the role and relevance of the different participants involved in heterochromatin silencing at *mat*, we have used genetic and biochemical approaches combining the expression of Atf1 derivatives with the insertion of different reporters inside the *mat locus* of fission yeast. *ura4* mRNA repression and mating capacity of the original *KΔ*::*ura4*^*+*^ *ura4-off* strain, kindly provided by the Grewal lab, was unexpectedly weak, but *atf1* deletion was still capable of exacerbating both defects in this background. One of the key proposals of our study is the role of Atf1 phosphorylation on the recruitment of SHREC to promote silencing, but other histone modifiers such as Clr6 or Clr4 are known to affect heterochromatin silencing and would be interesting to test whether Atf1 phosphorylation affects their recruitment as well. Regarding *mat2/3* switching, our experiments suggest that Atf1 phosphorylation may affect the recruitment to heterochromatin of the recombination complex including Swi2 and Swi5, and this will have to be experimentally validated with ChIP analysis.

## STAR★Methods

### Key resources table

**Table:** 

REAGENT or RESOURCE	SOURCE	IDENTIFIER
**Antibodies**		

Anti-Atf1 polyclonal	Laboratory made	(Sanso et al., 2008)
Anti-HA monoclonal	Laboratory made	12CA5
Anti-Myc	Merck Life Science	C3956; RRID:AB_439680
Anti-Sty1 monoclonal	Laboratory made	(Calvo et al., 2009)
Anti-H3K9me2	Abcam	Ab1220; RRID:AB_449854
Anti-H3k9ac	Millipore	07-352; RRID:AB_310544

**Chemicals, peptides, and recombinant proteins**		

5-FOA	Toronto Research Chemicals Inc	220141-70-8
Light Cycler 480 SYBR Green I Master	Roche	04707516001
Hydrogen peroxide	Sigma	H1009
Formaldehyde 37 %	Sigma	1.04002
Glycine	Sigma	200-272-2
TCA	VWR	1,00807,0250
Iodine	Sigma	I-3380

**Critical commercial assays**		

Reverse Transcription System of Applied Biosystem	Thermo Fisher Scientific	4374966

**Deposited data**		

Raw data of images	This paper; Mendeley Data	https://doi.org/10.17632/tc2pd8fjpk.1

**Experimental models: Organisms/strains**		

Yeast strains	See Table S1	N/A

**Oligonucleotides**		

Oligonucleotides	See Table S2	N/A

**Recombinant DNA**		

p428’	*psty1::HA-atf1*	(Salat-Canela et al., 2017)
p428’.10M	*psty1::HA-atf1.10M*	(Salat-Canela et al., 2017)
p428’.10D	*psty1::HA-atf1.10D*	(Salat-Canela et al., 2017)
p428’.bZIP	*psty1::HA-atf1*^*bZIP*^	This study
p482’	*psty1::HA-atf1*^*Δ6P Δinter*^	This study
p483’	*psty1::HA-atf1*^*Δinter*^	This study
p484’	*psty1::HA-atf1*^*Δ6P*^	This study
*pFA6a-natMX6*	*natMX6*	(Bahler et al., 1998)
*pFA6a-13myc::kanMX6*	*13myc::kanMX6*	(Bahler et al., 1998)
*pFA6a-13myc::natMX6*	*13myc::natMX6*	(Bahler et al., 1998)

**Software and algorithms**		

Adobe Illustrator 2021	Adobe	N/A
FiJi-ImageJ	NIH	https://imagej.net/software/fiji/
Gen5 software	Biotek	N/A
GraphPad Prism (6.0c)	GraphPad Software	https://www.graphpad.com/

### Resource availability

#### Lead contact

Further information and requests for resources and new reagents generated should be directed to and will be fulfilled by the lead contact, Elena Hidalgo (elena.hidalgo@upf.edu).

#### Materials availability

Plasmids and strains generated are available upon request to the lead contact.

### Experimental model and subject details

Fission yeast strains were grown in rich medium (YE5S) or minimal medium (MM) at 30 °C as described previously (Alfa et al., 1993). The genotypes of strains used in this study are shown in Table S1.

### Method details

#### Yeast strains, plasmids and growth conditions

To express wild-type HA-Atf1, HA-Atf1 phospho mutants and HA-Atf1 truncated mutants under the control of the constitutive *sty1* promoter in the different heterochromatin silencing reporter systems, strains SPJ236, SPJ266 and SPJ256 (Jia et al., 2004a) were transformed with the *leu1-32* integrative plasmids p428′ and p428’.10M and p428’.10D phospho mutant derivatives (Salat-Canela et al., 2017), as well as with p428′ truncated derivatives, generated as follows: p428’.bZIP lacks codons 1 to 365, p482’ (HA-Atf1^Δ6P Δinter^) lacks codons 135 to 365, p483’ (HA-Atf1 ^Δinter^) does not contain codons 254 to 365 and p484’ (HA-Atf1^Δ6P^) lacks codons135 to 227. To delete *sty1* in all *KΔ::ura4*^*+*^*(off)* backgrounds, we transformed the LS48, LS48.10M, LS48.10D, LS60, LS61 and LS62 strains with linear fragments containing *sty1*::*natMX6*, obtained by PCR amplification using *sty1* ORF-specific primers and plasmid *pFA6a-natMX6* as a template (Bahler et al., 1998). To delete *clr1, clr2, mit1, chp2* and *clr3* in *KΔ::ura4*^*+*^*(off) HA-atf1* background, the strain LS48 was transformed with linear fragments containing each *ORF*::*natMX6,* obtained by PCR amplification using ORF-specific primers and plasmid *pFA6a-natMX6* as a template. Finally, in order to tag Clr3 with Myc in *KΔ::ura4*^*+*^*(off) HA-atf1* backgrounds, we transformed the corresponding strains with a linear fragment containing the 3′ end of *clr3* fused to *myc::kanMX6* or *myc::natMX6* , obtained by PCR amplification using *clr3* specific primers and the plasmids *pFA6a-13myc::kanMX6* or *pFA6a-13myc::natMX6* (Bahler et al., 1998).

#### FOA survival assays

FOA survival assays were performed as described before (Garcia et al., 2014), with some modifications. Briefly, strains were grown at 30 °C in YE5S medium until they reached an OD_600_ of 0.5. The same number of cells (10^5^–10) in 3 μL was spotted on YE5S plates, in MM plates lacking uracil, or in YE plates containing the appropriated amount of the required supplements (Adenine, Histidine and Leucine, final concentration of 0.25 g/L) and half of the usual quantity of Uracil (final concentration of 0.125 g/L) plus a final concentration of 1 mg/mL fluoroorotic acid (FOA). The spots were allowed to dry and the plates were incubated at 30 °C for 2–4 days.

#### RNA analysis by reverse transcriptase quantitative PCR (RT-qPCR)

Total RNA was extracted from cultures of cells at an OD_600_ of 0.5 by standard hot-phenol method, as described before (Castillo et al., 2003). Reverse transcription and cDNA quantification was performed as previously described (Sanchez-Mir et al., 2020). Briefly, purified RNA was treated with DNase I and reverse-transcribed to cDNA using Reverse Transcription System of Applied Biosystems (Thermo Fisher Scientific), following the manufacturer’s instructions. cDNA was quantified by real-time quantitative PCR on Light Cycler II using Light Cycler 480 SYBR Green I Master (Roche). The error bars (standard deviation, SD) were calculated from at least three biological replicates, as indicated, and *act1* gene was used as a control for normalization. Fold induction was calculated comparing the value of each strain and condition to that of the wild-type strain. Primers used are listed in Table S2.

#### Chromatin immuno-precipitation (ChIP)

Cells were grown in YE medium and chromatin isolation and immunoprecipitation were carried out as previously described (Sanso et al., 2011), with minor modifications. Briefly, cells from 50-mL cultures were cross-linked with 1% formaldehyde for 10 (Clr3-Myc), 15 (Atf1-HA and H3K4me3) or 20 min (H3K9me2 and H3K9ac). Crosslinking was stopped with 125 mM glycine and after lysis of pellets with a bead beater, the lysates were sonicated in order to obtain chromatin fragments of ∼400 bp average size. Once the chromatin was isolated, it was immuno-precipitated with specific antibodies [5 μL of anti-HA antiserum (12CA5; house-made), 1 μL of anti-Myc (Merck Life Science, C3956), 1 μL of anti-H3K9me2 (Abcam, Ab1220) or 1 μL of anti-H3K9ac (Millipore) overnight at 4 °C rotating. Beads were washed, DNA was eluted and formaldehyde cross-linking was reversed. After protein digestion and chromatin extraction, DNA was amplified by quantitative PCR using Light Cycler 480 SYBR Green I Master (Roche). The error bars (SD) were calculated from at least three biological replicates, unless indicated otherwise. Primers from a mitochondrial DNA region or from *act1* ORF gene was used as a negative control, as indicated, and they are listed in Table S2.

#### Mating efficiency assay

Homothallic *h*^*90*^ strains were used to determine mating efficiency. Cells were grown to mid-log phase in standard MM and then shifted for 24 hours to MM without nitrogen. The number of unmated (or vegetative) cells, zygotes and asci was counted under light microscopy. The efficiency of conjugation or sporulation was calculated with the following ratio: 2× (number of zygotes or asci, respectively)/(total number of vegetative cells + 2× number of zygotes or asci). At least 200 cells from each biological triplicate were counted, and the mean ± SD was calculated.

#### Quantification of the *P/M* ratio in cell populations by qPCR from genomic DNA

We performed small scale purification of genomic DNA as described (Looke et al., 2011), with some modifications. ∼10^7^–10^8^ cells freshly thawed and grown in a YE agar plate were resuspended in 100 μL of a freshly prepared lithium acetate 0.2 M, 1% SDS solution. After incubating 5 min at 65°C, 300 μL 100% ethanol was added to the sample and centrifuged 3 min at full speed. The pellet was washed with 70% ethanol, and resuspended in H_2_O. Then, 2 μL of this suspension were used as template for qPCR. Two different sets of primers were used, sharing a common forward primer hybridizing at *mat1* and each of them with the specific reverse primer for *mat2* or *mat3*. A control mix containing 50% of genomic DNA of strain 972 (*h*^*-*^) and 50% of genomic DNA of strain 975 (*h*^*+*^) was used in each qPCR experiment to calculate the efficiency of the primers and adjust the results. Again, primers are listed in Table S2.

#### Sporulation capacity assay by iodine staining

Frozen cells were freshly thawed on YE plates for 2 days. Then they were resuspended in liquid MM lacking nitrogen, and after cell counting 10^5^ cells were dropped in MM without nitrogen plates supplemented with glutamate at a final concentration of 6 mM. After three days of growing at 25°C, plates were stained with iodine vapors and photographed immediately.

#### Experiment of evolution of HA-Atf1-and HA-Atf1.10M-expressing strains

Strain SPJ236 (Jia et al., 2004a) was freshly transformed with plasmids p428′ and p428.10M’ (Salat-Canela et al., 2017). Colonies selected in MM plates by the loss of leucine auxotrophy were expanded on strikes, PCR-checked for the proper insertion of the Atf1-containing plasmids and immediately frozen at −80°C. These strains were thawed and grown in MM plates for 24 h, and then inoculated in MM liquid cultures, at a starting OD_600_ of ∼0.015. Every 5 generations, 10 mL of cultures at an OD_600_ of 0.5 (equivalent to ∼10^8^ cells) were centrifuged, and cell pellets were frozen; in parallel, the cultures were diluted to a starting OD_600_ of ∼0.015 to reach an OD_600_ of 0.5 after 5 generations. Once all the cell pellets were collected, they were all thawed and processed to measure the *P/M* cells ratio as described above.

#### *S. pombe* TCA extracts and immunoblot analysis

Modified TCA extracts were prepared as described previously (Vivancos et al., 2005). Atf1 was immuno-detected with polyclonal anti-Atf1 (Sanso et al., 2008). Anti-Sty1 polyclonal antibody (Calvo et al., 2009) was used as loading control.

### Quantification and statistical analysis

Unless otherwise stated, all experiments were performed at least three times and representative experiments were shown. Data are presented as mean ± standard deviation (SD); ∗p < .05; ∗∗p < .01; ∗∗∗p < .001; ∗∗∗∗p < .0001 (Student’s t test). Graphs and statistical analysis were performed with Prism (GraphPad Software). Details of the statistical test used in each case can be found in the figure legend.

## Data Availability

• All images included in the main and supplemental figures have been deposited at Mendeley and are publicly available as of the date of publication. The DOI is listed in the key resources table. • Any additional information required to reanalyze the data reported in this work paper is available from the lead contact upon request.

## Supporting Citations

The following reference appears in the Supplemental Information: Leupold, 1970
