# Supplementary material for: A stress-blinded Atf1 can fully assemble heterochromatin in a RNAi-independent minimal mat locus but impairs directionality of mat2/3 switching
Source: iScience. 2022 Aug 2;25(8):104820. doi: 10.1016/j.isci.2022.104820 (PMC9389250; doi:10.1016/j.isci.2022.104820)
Supplement: Document S1. Figures S1–S4 and Tables S1 and S2 [file mmc1.pdf]

iScience, Volume *25*

Supplemental information

A stress-blinded Atf1 can fully assemble heterochromatin in a RNAi-independent minimal mat locus but impairs directionality of *mat2/3* switching

Rodrigo Fraile, Laura Sánchez-Mir, Guillem Murciano-Julià, José Ayté, and Elena Hidalgo

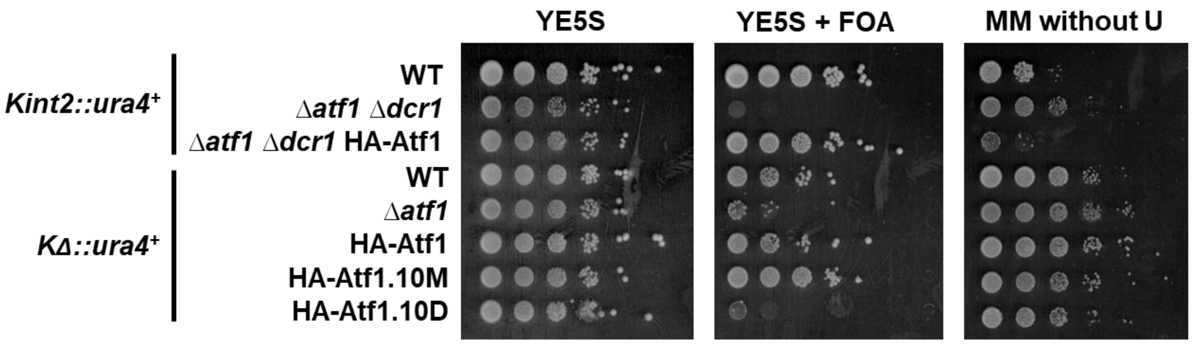

Figure S1. Role of Atf1 and RNAi in heterochromatin at *Kint2::ura4*$^{+}$ and *K*Δ*::ura4*$^{+}$ *ura4-off* (Related to Figure 1)

Serial dilutions of exponentially growing cultures of the indicated strains were spotted on YE5S plates with or without FOA (1 mg/ml), or on MM plates lacking uracil (but containing adenine, histidine and leucine).

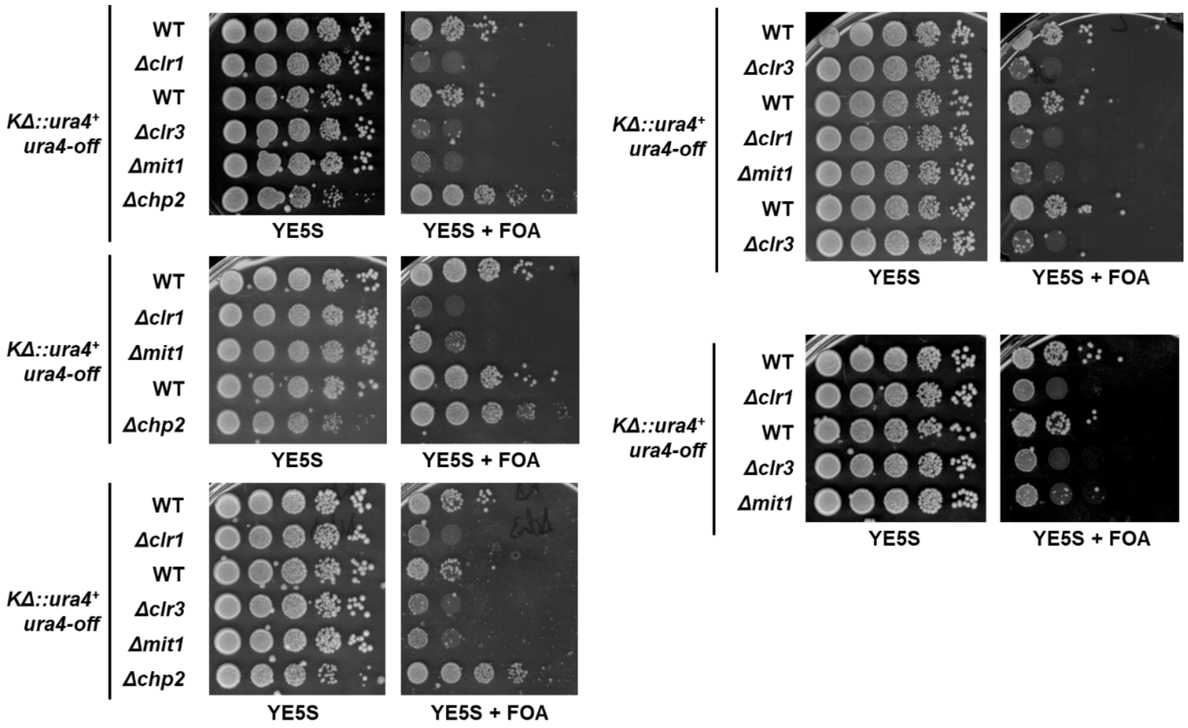

Figure S2. The SHREC complex is essential to silence heterochromatin at *mat* (Related to Figure 3)

Role of SHREC in heterochromatin at *K*Δ*::ura4*$^{+}$*ura4-off.* The indicated strains were analyzed as in Figure 1D.

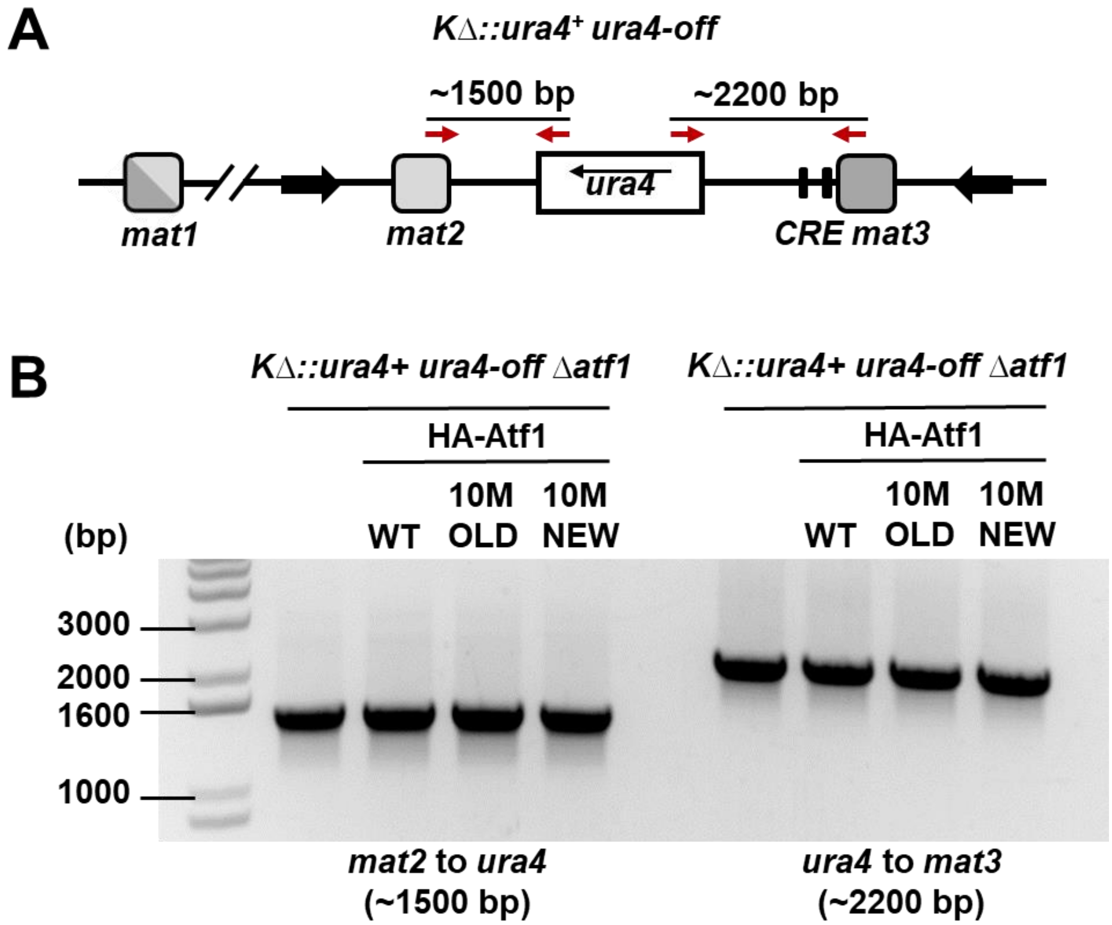

Figure S3. Analysis of the relative position of *mat2* and *mat3* in different *KΔ::ura4*$^{+}$ *ura4-off* Δ*atf1* derivatives (Related to Figure 5)

(A) Scheme of the *mat* locus in *KΔ::ura4*$^{+}$ *ura4-off* background, with the relative position of the *mat2* and *mat3* genes flanking the inverted *ura4* cassette. Primers to amplify the *mat2* to *ura4* (~1500 bp) and *ura4* to *mat3* (~2200 bp) regions are indicated in red.

(B) Genomic DNA of the indicated strains was PCR-amplified with the primers OLEH-AA62 and OLEH-AA64 (*mat2* to *ura4* region) and OLEH-AA65 and OLEH-AA63 (*ura4* to *mat3* region) and loaded in an agarose gel. The sizes of the molecular weight markers are indicated.

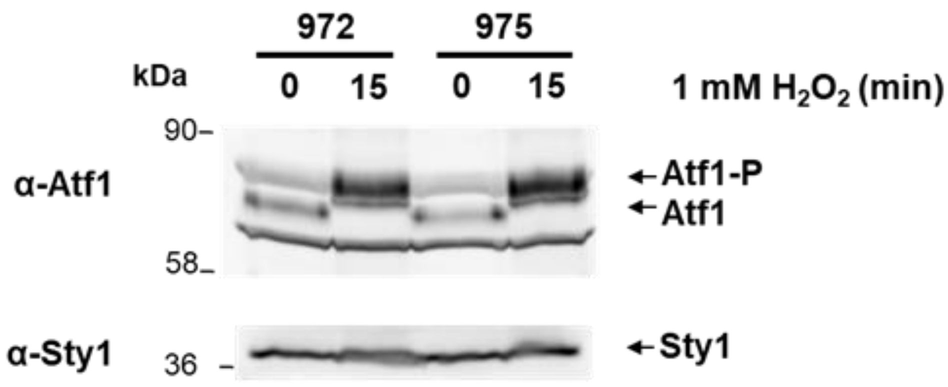

Figure S4. *h*$^+$ and *h*$^-$ cells do not display differences in the levels of phosphorylation of Atf1 (Related to Figure 4)

Cultures of strains 972 (*h*$^-$) and 975 (*h*$^+$) were treated or not with 1mM H_2O_2 for 15 min. TCA protein extracts were prepared and analyzed by Western blotting using polyclonal antibodies anti-Atf1. Antibodies against Sty1 were used as a loading control.

Table S1. Strains used in this study (Related to STAR Methods)

Strain	Genotype	Origin
**SPJ83**	*h^{90} leu1-32 his2 ura4DS/E ade6-210 Kint2::ura4^{+}*	(Jia et al., 2004)
**SPG1398**	*h^{90} leu1-32 his2 ura4DS/E ade6-210 Kint2::ura4^{+} dcr1::kanMX6*	(Jia et al., 2004)
**SPJ266**	*h^{90} leu1-32 his2 ura4DS/E ade6-216 Kint2::ura4^{+} atf1::kanMX6*	(Jia et al., 2004)
**LS49**	*h^{90} leu1-32 his2 ura4DS/E ade6-216 Kint2::ura4^{+} atf1::kanMX6 psty1'::HA-atf1::leu1^{+}*	This work
**LS49.10M**	*h^{90} leu1-32 his2 ura4DS/E ade6-216 Kint2::ura4^{+} atf1::kanMX6 psty1'::HA-atf1.10M::leu1^{+}*	This work
**LS49.10D**	*h^{90} leu1-32 his2 ura4DS/E ade6-216 Kint2::ura4^{+} atf1::kanMX6 psty1'::HA-atf1.10D::leu1^{+}*	This work
**LS49.bZIP**	*h^{90} leu1-32 his2 ura4DS/E ade6-216 Kint2::ura4^{+} atf1::kanMX6 psty1'::HA-atf1.bZIP::leu1^{+}*	This work
**SPJ256**	*h^{90} leu1-32 his2 ura4DS/E ade6-216 Kint2::ura4^{+} atf1::kanMX6 dcr1::kanMX6*	(Jia et al., 2004a)
**LS51**	*h^{90} leu1-32 his2 ura4DS/E ade6-216 Kint2::ura4^{+} atf1::kanMX6 dcr1::kanMX6 psty1'::HA-atf1::leu1^{+}*	This work
**LS51.10M**	*h^{90} leu1-32 his2 ura4DS/E ade6-216 Kint2::ura4^{+} atf1::kanMX6 dcr1::kanMX6 psty1'::HA-atf1.10M::leu1^{+}*	This work
**LS51.10D**	*h^{90} leu1-32 his2 ura4DS/E ade6-216 Kint2::ura4^{+} atf1::kanMX6 dcr1::kanMX6 psty1'::HA-atf1.10D::leu1^{+}*	This work
**LS51.bZIP**	*h^{90} leu1-32 his2 ura4DS/E ade6-216 Kint2::ura4+ atf1::kanMX6 dcr1::kanMX6 psty1'::HA-atf1.bZIP::leu1^{+}*	This work
**SPG1161**	*h^{90} leu1-32 ura4DS/E ade6-210 KΔ::ura4^{+}(off)*	(Jia et al., 2004a)
**SPJ236**	*h^{90} leu1-32 ura4DS/E ade6-216 KΔ::ura4^{+}(off) atf1::kanMX6*	(Jia et al., 2004a)
**LS48**	*h^{90} leu1-32 ura4DS/E ade6-216 KΔ::ura4^{+}(off) atf1::kanMX6 psty1'::HA-atf1::leu1+*	This work
**LS48.10M**	*h^{90} leu1-32 ura4DS/E ade6-216 KΔ::ura4^{+}(off) atf1::kanMX6 psty1'::HA-atf1.10M::leu1^{+}*	This work
**LS48.10D**	*h^{90} leu1-32 ura4DS/E ade6-216 KΔ::ura4^{+}(off) atf1::kanMX6 psty1'::HA-atf1.10D::leu1^{+}*	This work
**LS48.bZIP**	*h^{90} leu1-32 ura4DS/E ade6-216 KΔ::ura4^{+}(off) atf1::kanMX6 psty1'::HA-atf1.bZIP::leu1^{+}*	This work
**LS66**	*h^{90} leu1-32 his2 ura4DS/E ade6-216 Kint2::ura4+ atf1::kanMX6 psty1'::HA-atf1$^{\Delta 6P\ \Delta inter}$::leu1^{+}*	This work
**LS67**	*h^{90} leu1-32 his2 ura4DS/E ade6-216 Kint2::ura4^{+} atf1::kanMX6 psty1'::HA-atf1$^{\Delta inter}$::leu1^{+}*	This work
**LS68**	*h^{90} leu1-32 his2 ura4DS/E ade6-216 Kint2::ura4^{+} atf1::kanMX6 psty1'::HA-atf1$^{\Delta 6P}$::leu1^{+}*	This work
**LS63**	*h^{90} leu1-32 his2 ura4DS/E ade6-216 Kint2::ura4^{+} atf1::kanMX6 dcr1::kanMX6 psty1'::HA-atf1$^{\Delta 6P\ \Delta inter}$::leu1^{+}*	This work
**LS64**	*h^{90} leu1-32 his2 ura4DS/E ade6-216 Kint2::ura4^{+} atf1::kanMX6 dcr1::kanMX6 psty1'::HA-atf1 $^{\Delta inter}$::leu1^{+}*	This work
**LS65**	*h^{90} leu1-32 his2 ura4DS/E ade6-216 Kint2::ura4^{+} atf1::kanMX6 dcr1::kanMX6 psty1'::HA-atf1$^{\Delta 6P}$::leu1^{+}*	This work
**972**	*h^{-}*	(Leupold, 1970)
**975**	*h^{+}*	(Leupold, 1970)
**LS60**	*h^{90} leu1-32 ura4DS/E ade6-216 KΔ::ura4^{+}(off) atf1::kanMX6 psty1'::HA-atf1$^{\Delta 6P\ \Delta inter}$::leu1^{+}*	This work
**LS61**	*h^{90} leu1-32 ura4DS/E ade6-216 KΔ::ura4^{+}(off) atf1::kanMX6 psty1'::HA-atf1 $^{\Delta inter}$::leu1^{+}*	This work
**LS62**	*h^{90} leu1-32 ura4DS/E ade6-216 KΔ::ura4^{+}(off) atf1::kanMX6 psty1'::HA-atf1 $^{\Delta 6P}$::leu1^{+}*	This work

**LS99**	*h^{90} leu1-32 ura4DS/E ade6-216 KΔ::$ura4^{+}$(off) sty1::natMX6*	This work
**LS79**	*h^{90} leu1-32 ura4DS/E ade6-216 KΔ::$ura4^{+}$(off) atf1::kanMX6 sty1::natMX6 psty1'::HA-$atf1^{\Delta 6P\ \Delta inter}$::$leu1^{+}$*	This work
**LS80**	*h^{90} leu1-32 ura4DS/E ade6-216 KΔ::$ura4^{+}$(off) atf1::kanMX6 sty1::natMX6 psty1'::HA-$atf1^{\Delta inter}$::$leu1^{+}$*	This work
**LS81**	*h^{90} leu1-32 ura4DS/E ade6-216 KΔ::$ura4^{+}$(off) atf1::kanMX6 sty1::natMX6 psty1'::HA-$atf1^{\Delta 6P}$::$leu1^{+}$*	This work
**LS78**	*h^{90} leu1-32 ura4DS/E ade6-216 KΔ::$ura4^{+}$(off) atf1::kanMX6 sty1::natMX6 psty1'::HA-atf1::$leu1^{+}$*	This work
**LS78.10M**	*h^{90} leu1-32 ura4DS/E ade6-216 KΔ::$ura4^{+}$(off) atf1::kanMX6 sty1::natMX6 psty1'::HA-atf1.10M::$leu1^{+}$*	This work
**LS78.10D**	*h^{90} leu1-32 ura4DS/E ade6-216 KΔ::$ura4^{+}$(off) atf1::kanMX6 sty1::natMX6 psty1'::HA-atf1.10D::$leu1^{+}$*	This work
**RF62**	*h^{90} leu1-32 ura4DS/E ade6-216 KΔ::$ura4^{+}$(off) atf1::kanMX6 psty1'::HA-atf1::$leu1^{+}$ clr1::natMX6*	This work
**RF63**	*h^{90} leu1-32 ura4DS/E ade6-216 KΔ::$ura4^{+}$(off) atf1::kanMX6 psty1'::HA-atf1::$leu1^{+}$ mit1::natMX6*	This work
**RF65**	*h^{90} leu1-32 ura4DS/E ade6-216 KΔ::$ura4^{+}$(off) atf1::kanMX6 psty1'::HA-atf1::$leu1^{+}$ chip2::natMX6*	This work
**RF116**	*h^{90} leu1-32 ura4DS/E ade6-216 KΔ::$ura4^{+}$(off) atf1::kanMX6 psty1'::HA-atf1::$leu1^{+}$ clr3::natMX6*	This work
**LS74**	*h^{90} leu1-32 ura4DS/E ade6-216 KΔ::$ura4^{+}$(off) atf1::kanMX6 psty1'::HA-atf1::$leu1^{+}$ Clr3-myc::kanMX6*	This work
**LS75**	*h^{90} leu1-32 ura4DS/E ade6-216 KΔ::$ura4^{+}$(off) atf1::kanMX6 psty1'::HA-$atf1^{\Delta 6P\ \Delta inter}$::$leu1^{+}$ Clr3-myc::kanMX6*	This work
**LS76**	*h^{90} leu1-32 ura4DS/E ade6-216 KΔ::$ura4^{+}$(off) atf1::kanMX6 psty1'::HA-$atf1^{\Delta inter}$::$leu1^{+}$ Clr3-myc::kanMX6*	This work
**LS77**	*h^{90} leu1-32 ura4DS/E ade6-216 KΔ::$ura4^{+}$(off) atf1::kanMX6 psty1'::HA-$atf1^{\Delta 6P}$::$leu1^{+}$ Clr3-myc::kanMX6*	This work
**JA209**	*h^{90}*	Laboratory stock
**RF121.10M**	*h^{90} leu1-32 ura4DS/E ade6-216 KΔ::$ura4^{+}$(off) atf1::kanMX6 psty1'::HA-atf1.10M::$leu1^{+}$ Clr3-myc::natMX6*	This work
**RF121.10D**	*h^{90} leu1-32 ura4DS/E ade6-216 KΔ::$ura4^{+}$(off) atf1::kanMX6 psty1'::HA-atf1.10D::$leu1^{+}$ Clr3-myc::natMX6*	This work
**RF122**	*h^{90} leu1-32 ura4DS/E ade6-216 KΔ::$ura4^{+}$(off) atf1::kanMX6 psty1'::HA-atf1::$leu1^{+}$ clr2::natMX6*	This work

Table S2. Primers used in this study for RT-qPCR and ChIP experiments (Related to STAR Methods)

Primer	Sequence	Gene	Application
OLEH-T97	5´-GCTACAATATGCATCTGGTGTGTAC-3´	*ura4* F	RT-qPCR
OLEH-T98	5´-TCTTTGAGGCCTTGTATAATACCCT-3´	*ura4* R	RT-qPCR
OLEH-N7	5´-ACACTAGACGAAGAGACGCATGTAT-3´	*ste11* F	RT-qPCR
OLEH-N8	5´-ACCGAACTTTTATCTACGCCTTATT-3´	*ste11* R	RT-qPCR
OLEH-H48	5´-TGCCCCTAGAGCTGTATTCC-3´	*act1* F	RT-qPCR/ChIP
OLEH-H49	5´-TTTGAGCTTCATCACCAACG-3´	*act1* R	RT-qPCR/ChIP
OLEH-P83	5´-CATATCAATTACAAACATATTGTTTGCTGC-3´	*CRE* F	ChIP
OLEH-P84	5´-GCTTCATGTTAAGTTGATTGGTGTAATTAG-3´	*CRE* R	ChIP
J2026	5'-TGGTAGAGCAAGTCACTGTTAATGA-3'	mtDNA F	ChIP
J2027	5'-TGGTAGAGCAAGTCACTGTTAATGA-3'	mtDNA R	ChIP
OLEH-U23	5´-AGAAGAGAGAGTAGTTGAAG-3´	*mat1 comm. F*	RT-qPCR
OLEH-U24	5´-ACGGTAGTCATCGGTCTTCC-3´	*mat1-P R*	RT-qPCR
OLEH-U25	5´-TACGTTCAGTAGACGTAGTG-3´	*mat1-M R*	RT-qPCR
OLEH-AA62	5’-GGGCCAATTCTACGAAGTTTTAAGG-3´	*mat2 F*	PCR-agar.gels
OLEH-AA63	5´-CTAACATCAAATTACAACTTAAACG-3´	*mat3 R*	PCR-agar.gels
OLEH-AA64	5´-CTATGTACAAAGCCAATGAAAGATG-3´	*ura4 R*	PCR-agar.gels
OLEH-AA65	5´-GCATACATATAGCCAGTGGGATTTG-3´	*ura4 F*	PCR-agar.gels
